# Supplementary material for: Streptavidins Coordinate Biotin Sequestration and Self‐Resistance Within a Biotin‐Pathway Antibiotic Network
Source: Adv Sci (Weinh). 2026 May 23:e23813. Online ahead of print. doi: 10.1002/advs.202523813 (PMC13336041; doi:10.1002/advs.202523813)
Supplement: Supplementary file 1 — Supporting File: advs75794‐sup‐0001‐SuppMat.docx. [file ADVS-9999-e23813-s001.docx]

**Streptavidins Coordinate Biotin Sequestration and Self-Resistance within a Biotin-Pathway Antibiotic Network**

Sumire Kurosawa^1,2,3^†, Jingjun Mo^1,2,3^†, Xiaotong Diao^4^†, Feng Xie^1,2^†, Tingting Wang^1,2^, Haowen Zhao^1,2,3^, Dalei Wu^4^, Rolf Müller^1,2,3,5,6^*, Chengzhang Fu^1,2,6^*

^1^Helmholtz Institute for Pharmaceutical Research Saarland (HIPS), Helmholtz Centre for Infection Research (HZI), 66123 Saarbrücken, Germany

^2^Helmholtz International Lab for Anti-Infectives, Helmholtz Center for Infection Research, 38124 Braunschweig, Germany

^3^Saarland University, Department of Pharmacy, Campus Building E8.1, 66123 Saarbrücken, Saarland, Germany.

^4^Helmholtz International Lab for Anti-Infectives, State Key Laboratory of Microbial Technology, Shandong University, Qingdao 266237, China

^5^German Centre for Infection Research (DZIF), 38124 Braunschweig, Germany

^6^PharmaScienceHub, Saarbrücken 66123, Germany.

†These authors contributed equally to this work.

*Corresponding author. Email: chengzhang.fu@helmholtz-hips.de; rolf.mueller@helmholtz-hips.de

**Contents**

[**Supplementary Figures** 4](#_Toc226626062)

[**Supplementary Figure 1** Verification of the mobilization of *Svi7-1* from the chromosome of *S. virginiae* by single-plasmid ACTIMOT. 4](#_Toc226626063)

[**Supplementary Figure 2** Validation of the mobilized TDR *Svi7-1* via mapping the short-read sequencing data to pCap-*Svi7-1*. 4](#_Toc226626064)

[**Supplementary Figure 3** Chemical structures of all detected compounds in *S. virginiae* ACTIMOT mutant. 5](#_Toc226626065)

[**Supplementary Figure 4** High-resolution mass spectra (HRMS) of purified compounds. 5](#_Toc226626066)

[**Supplementary Figure 5** Determination of compounds **1** and **8** using UPLC-HRMS analysis. 6](#_Toc226626067)

[**Supplementary Figure 6** UPLC-MS analysis of *mkp* gene deletion mutants. 6](#_Toc226626068)

[**Supplementary Figure 7** Isotope-labeling analysis for α-methyl-KAPA (**5**). 7](#_Toc226626069)

[**Supplementary Figure 8** *In vitro* conversion of α-methyl-KAPA (**5**) to α-methyldesthiobiotin (**3**) by *E. coli* BioAD. 7](#_Toc226626070)

[**Supplementary Figure 9** Detection of α-methyldesthiobiotin (**3**) in *S. virginiae* WT and its ACTIMOT mutant. 8](#_Toc226626071)

[**Supplementary Figure 10** Detection of α-methyldesthiobiotin (**3**) in *E. coli* BW25113 treated with α-methyl-KAPA (**5**). 8](#_Toc226626072)

[**Supplementary Figure 11** *In vitro* production of α-methyldesthiobiotin (**3)** from α-methyl-KAPA (**5**) using MdbA and MdbB. 9](#_Toc226626073)

[**Supplementary Figure 12** Marfey analysis of Ala-Glu dipeptide derived from Ala-ANDA (**10**). 10](#_Toc226626074)

[**Supplementary Figure 13** Isotope-labeling analysis for Ala-ANDA (**10**). 10](#_Toc226626075)

[**Supplementary Figure 14** Production of acidomycin (**1**) in *S. avidinii*-*kasO*p*-*acdA* and *S. albus* Del14::*kasO*p*-*Savi*-*acd*. 11](#_Toc226626076)

[**Supplementary Figure 15** Isotope-labeling analysis for acidomycin (**1**). 11](#_Toc226626077)

[**Supplementary Figure 16** Rescue assay for α-methyl-KAPA (**5**). 12](#_Toc226626078)

[**Supplementary Figure 17** *In vitro* inhibition assay of BioAD with α-methyl-KAPA (**5**). 13](#_Toc226626079)

[**Supplementary Figure 18** Structural analysis for streptavidin 2-acidomycin complex. 14](#_Toc226626080)

[**Supplementary Figure 19** COSY and Key HMBC correlations of **8**. 15](#_Toc226626081)

[**Supplementary Figure 20** COSY and Key HMBC correlations of α-methyl-KAPA (**5**). 15](#_Toc226626082)

[**Supplementary Figure 21** COSY and Key HMBC correlations of **9**. 15](#_Toc226626083)

[**Supplementary Figure 22** COSY and Key HMBC correlations of α-methyldesthiobiotin (**3**). 15](#_Toc226626084)

[**Supplementary Figure 23** COSY and Key HMBC correlations of ANDA (**6**). 15](#_Toc226626085)

[**Supplementary Figure 24** COSY and Key HMBC correlations of Ala-ANDA (**10**). 16](#_Toc226626086)

[**Supplementary Figure 25** COSY and Key HMBC correlations of (*E*)-**6***. 16](#_Toc226626087)

[**Supplementary Figure 26** COSY and Key HMBC correlations of (*Z*)-**6***. 16](#_Toc226626088)

[**Supplementary Figure 27** Conversion of Ala-Glu from Ala-ANDA (**10**). 16](#_Toc226626089)

[**Supplementary Figure 28** COSY and Key HMBC correlations of acidomycin (**1**). 16](#_Toc226626090)

[**Supplementary Figure 29** ^1^H NMR spectrum of **8** (500 MHz, D_2_O). 17](#_Toc226626091)

[**Supplementary Figure 30** HSQC spectrum of **8** (500 MHz, D_2_O). 18](#_Toc226626092)

[**Supplementary Figure 31** HMBC spectrum of **8** (500 MHz, D_2_O). 18](#_Toc226626093)

[**Supplementary Figure 32** COSY spectrum of **8** (500 MHz, D_2_O). 19](#_Toc226626094)

[**Supplementary Figure 33** ^1^H NMR spectrum of α-methyl-KAPA (**5**) (500 MHz, D_2_O). 20](#_Toc226626095)

[**Supplementary Figure 34** ^13^C NMR spectrum of α-methyl-KAPA (**5**) (125 MHz, D_2_O). 20](#_Toc226626096)

[**Supplementary Figure 35** HSQC spectrum of α-methyl-KAPA (**5**) (500 MHz, D_2_O). 21](#_Toc226626097)

[**Supplementary Figure 36** HMBC spectrum of α-methyl-KAPA (**5**) (500 MHz, D_2_O). 21](#_Toc226626098)

[**Supplementary Figure 37** COSY spectrum of α-methyl-KAPA (**5**) (500 MHz, D_2_O). 22](#_Toc226626099)

[**Supplementary Figure 38** ^1^H NMR spectrum of **9** (500 MHz, CD_3_OD). 23](#_Toc226626100)

[**Supplementary Figure 39** ^13^C NMR spectrum of **9** (125 MHz, CD_3_OD). 24](#_Toc226626101)

[**Supplementary Figure 40** HSQC spectrum of **9** (500 MHz, CD_3_OD). 24](#_Toc226626102)

[**Supplementary Figure 41** HMBC spectrum of **9** (500 MHz, CD_3_OD). 25](#_Toc226626103)

[**Supplementary Figure 42** COSY spectrum of **9** (500 MHz, CD_3_OD). 25](#_Toc226626104)

[**Supplementary Figure 43** ^1^H NMR spectrum of α-methyldesthiobiotin (**3**) (500 MHz, D_2_O). 26](#_Toc226626105)

[**Supplementary Figure 44** ^13^C NMR spectrum of α-methyldesthiobiotin (**3**) (125 MHz, D_2_O). 26](#_Toc226626106)

[**Supplementary Figure 45** HSQC spectrum of α-methyldesthiobiotin (**3**) (500 MHz, D_2_O). 27](#_Toc226626107)

[**Supplementary Figure 46** HMBC spectrum of α-methyldesthiobiotin (**3**) (500 MHz, D_2_O). 27](#_Toc226626108)

[**Supplementary Figure 47** COSY spectrum of α-methyldesthiobiotin (**3**) (500 MHz, D_2_O). 28](#_Toc226626109)

[**Supplementary Figure 48** ^1^H NMR spectrum of ANDA (**6**) (500 MHz, D_2_O). 29](#_Toc226626110)

[**Supplementary Figure 49** HSQC spectrum of ANDA (**6**) (500 MHz, D_2_O). 30](#_Toc226626111)

[**Supplementary Figure 50** HMBC spectrum of ANDA (**6**) (500 MHz, D_2_O). 30](#_Toc226626112)

[**Supplementary Figure 51** COSY spectrum of ANDA (**6**) (500 MHz, D_2_O). 31](#_Toc226626113)

[**Supplementary Figure 52** ^1^H NMR spectrum of Ala-ANDA (**10**) (500 MHz, CD_3_OD). 32](#_Toc226626114)

[**Supplementary Figure 53** ^13^C NMR spectrum of Ala-ANDA (**10**) (125 MHz, CD_3_OD). 32](#_Toc226626115)

[**Supplementary Figure 54** HSQC spectrum of Ala-ANDA (**10**) (500 MHz, CD_3_OD). 33](#_Toc226626116)

[**Supplementary Figure 55** HMBC spectrum of Ala-ANDA (**10**) (500 MHz, CD_3_OD). 33](#_Toc226626117)

[**Supplementary Figure 56** COSY spectrum of Ala-ANDA (**10**) (500 MHz, CD_3_OD). 34](#_Toc226626118)

[**Supplementary Figure 57** ^1^H NMR spectrum of (*E*)-**6*** (500 MHz, D_2_O). 35](#_Toc226626119)

[**Supplementary Figure 58** ^13^C NMR spectrum of (*E*)-**6*** (125 MHz, D_2_O). 35](#_Toc226626120)

[**Supplementary Figure 59** HSQC spectrum of (*E*)-**6*** (500 MHz, D_2_O). 36](#_Toc226626121)

[**Supplementary Figure 60** HMBC spectrum of (*E*)-**6*** (500 MHz, D_2_O). 36](#_Toc226626122)

[**Supplementary Figure 61** COSY spectrum of (*E*)-**6*** (500 MHz, D_2_O). 37](#_Toc226626123)

[**Supplementary Figure 62** ^1^H NMR spectrum of (*Z*)-**6*** (500 MHz, D_2_O). 38](#_Toc226626124)

[**Supplementary Figure 63** ^13^C NMR spectrum of (*Z*)-**6*** (125 MHz, D_2_O). 39](#_Toc226626125)

[**Supplementary Figure 64** HSQC spectrum of (*Z*)-**6*** (500 MHz, D_2_O). 39](#_Toc226626126)

[**Supplementary Figure 65** HMBC spectrum of (*Z*)-**6*** (500 MHz, D_2_O). 40](#_Toc226626127)

[**Supplementary Figure 66** COSY spectrum of (*Z*)-**6*** (500 MHz, D_2_O). 40](#_Toc226626128)

[**Supplementary Figure 67** ^1^H NMR spectrum of acidomycin (**1**) (500 MHz, CD_3_OD). 41](#_Toc226626129)

[**Supplementary Figure 68** ^13^C NMR spectrum of acidomycin (**1**) (125 MHz, CD_3_OD). 41](#_Toc226626130)

[**Supplementary Figure 69** HSQC spectrum of acidomycin (**1**) (500 MHz, CD_3_OD). 42](#_Toc226626131)

[**Supplementary Figure 70** HMBC spectrum of acidomycin (**1**) (500 MHz, CD_3_OD). 42](#_Toc226626132)

[**Supplementary Figure 71** COSY spectrum of acidomycin (**1**) (500 MHz, CD_3_OD). 43](#_Toc226626133)

[**Supplementary Tables** 44](#_Toc226626134)

[**Supplementary Table 1.** Bacterial strains used in this work. 44](#_Toc226626135)

[**Supplementary Table 3.** Primers used in this work. 47](#_Toc226626136)

[**Supplementary Table 4.** Gene annotation of the *sta*, *acd*, *mpk*, *acd*, and *mdb*. 50](#_Toc226626137)

[**Supplementary Table 5.** Data collection and refinement statistics. 52](#_Toc226626138)

[**Supplementary Table 6.** NMR data of **8** in CD_3_OD^1^. 53](#_Toc226626139)

[**Supplementary Table 7.** NMR data of α-methyl-KAPA (**5**) in D_2_O^1^. 53](#_Toc226626140)

[**Supplementary Table 8.** NMR data of **9** in CD_3_OD^1^. 54](#_Toc226626141)

[**Supplementary Table 9.** NMR data of α-methyldesthiobiotin (**3**) in D_2_O^1^. 54](#_Toc226626142)

[**Supplementary Table 10.** NMR data of ANDA (**6**) in D_2_O^1^. 55](#_Toc226626143)

[**Supplementary Table 11.** NMR data of Ala-ANDA (**10**) in CD_3_OD^1^. 55](#_Toc226626144)

[**Supplementary Table 12.** NMR data of (*E*)-**6*** in D_2_O^1^. 55](#_Toc226626145)

[**Supplementary Table 13.** NMR data of (*Z*)-(**6***) in D_2_O^1^. 56](#_Toc226626146)

[**Supplementary Table 14.** NMR data of acidomycin (**1**) in CD_3_OD^1^. 56](#_Toc226626147)

[**References** 57](#_Toc226626148)

Supplementary Figures


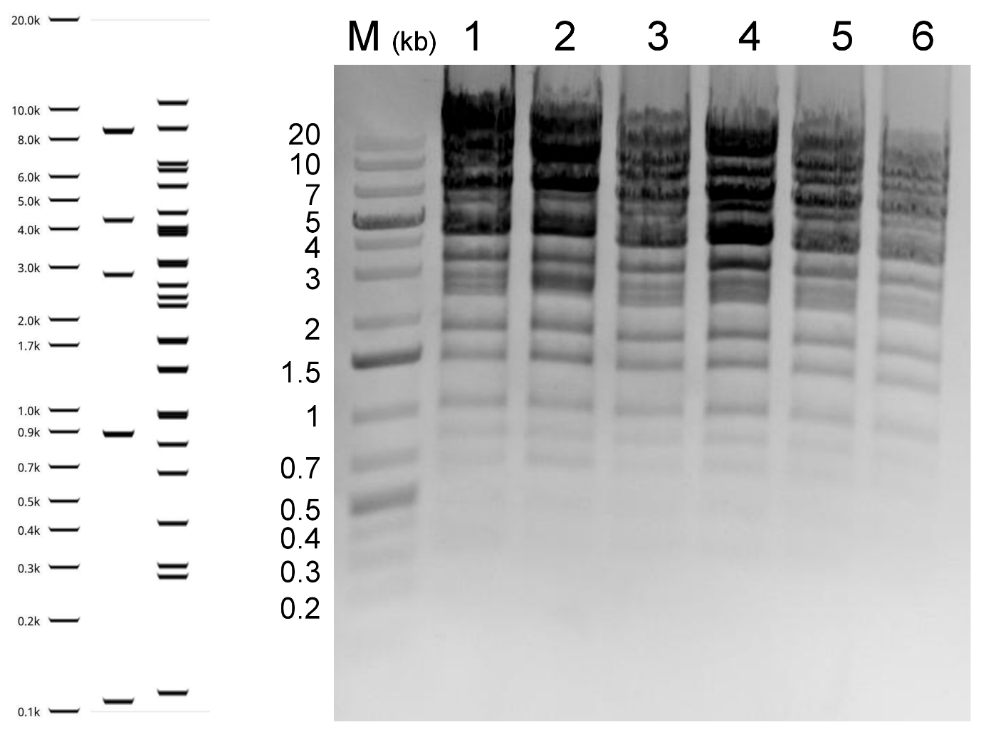


## **Supplementary** **Figure 1** Verification of the mobilization of *Svi7-1* from the chromosome of *S. virginiae* by single-plasmid ACTIMOT.

Six *E. coli* colonies obtained from two mobilization exconjugants were randomly picked for *Eco72*I digestion. The theoretical digestion pattern of pCap-101-Apr-SVI7-1-LR (left) and pCap-*Svi7-1* (right) is shown in the left panel. Lanes 1-3 and 4-6 are the plasmids recovered from two individual exconjugants, respectively. All of these plasmids show identical patterns to the theoretical one.


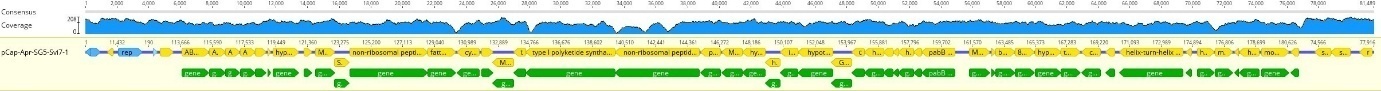


## **Supplementary Figure 2** Validation of the mobilized TDR *Svi7-1* via mapping the short-read sequencing data to pCap-*Svi7-1*.

**Supplementary Figure 3** Chemical structures of all detected compounds in *S. virginiae* ACTIMOT mutant.

KAPA: 7-keto-8-aminopelargonic acid; ANDA: 2-aminonona-5,7-diendioic acid.


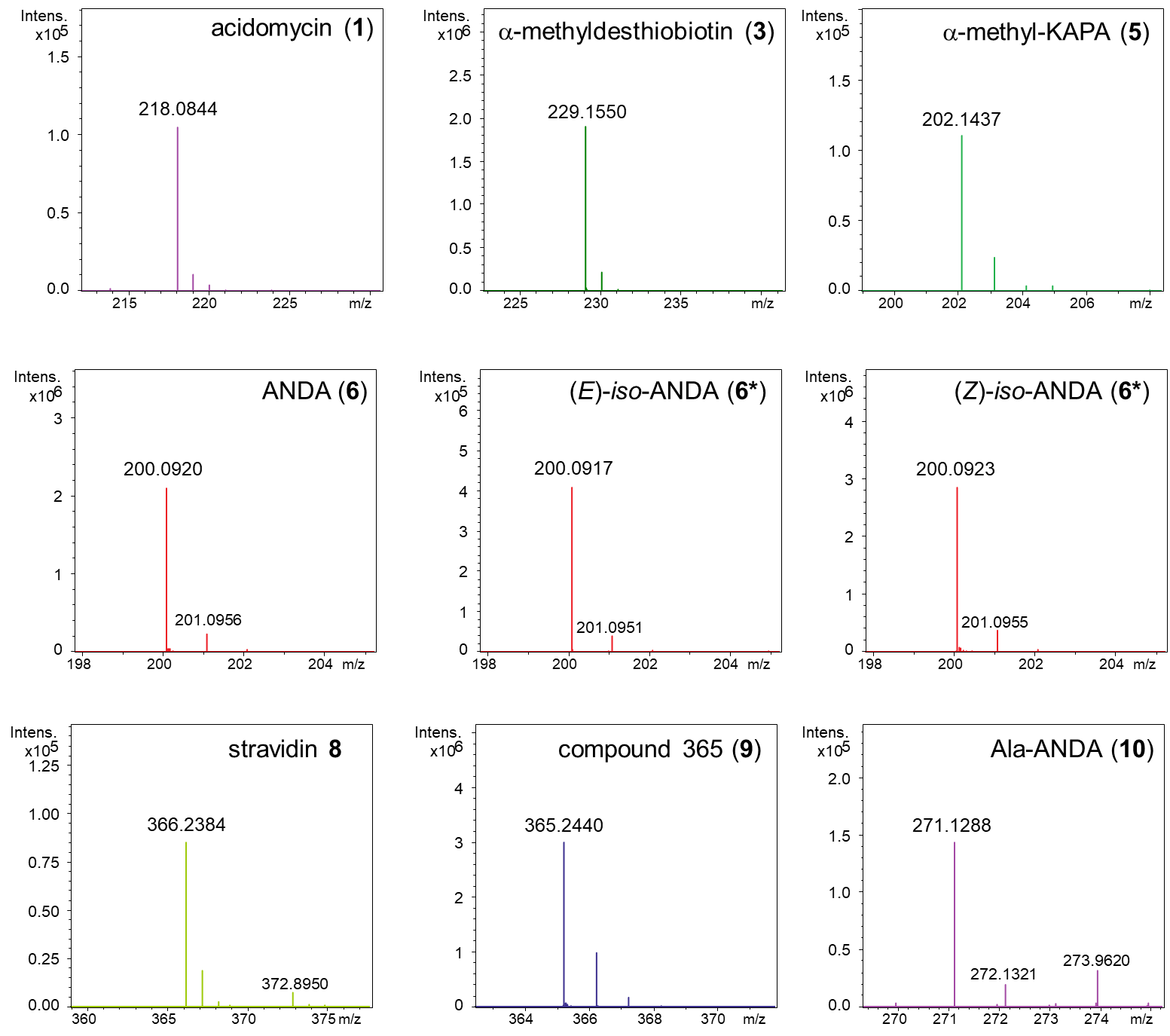


**Supplementary Figure 4** High-resolution mass spectra (HRMS) of purified compounds.

**
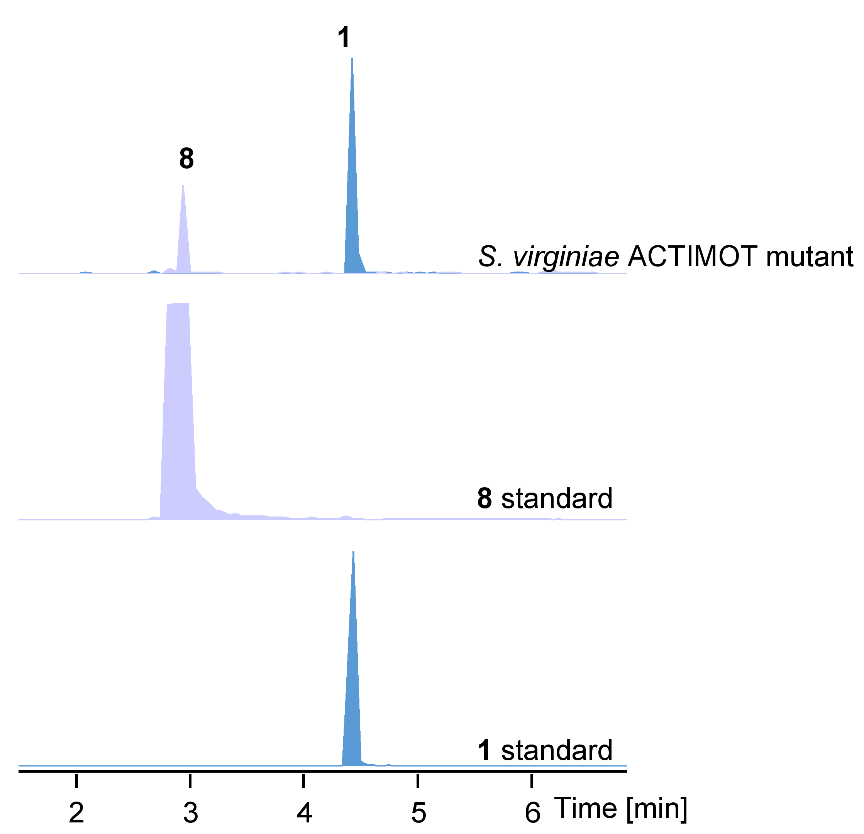
**

**Supplementary Figure 5** Determination of compounds **1** and **8** using UPLC-HRMS analysis.

Pure compounds isolated from *S. avidinii* were used as standards. Extracted ion chromatograms (EICs) for **1** (*m*/*z* 218.08) and **8** (*m*/*z* 366.24) are shown.

**
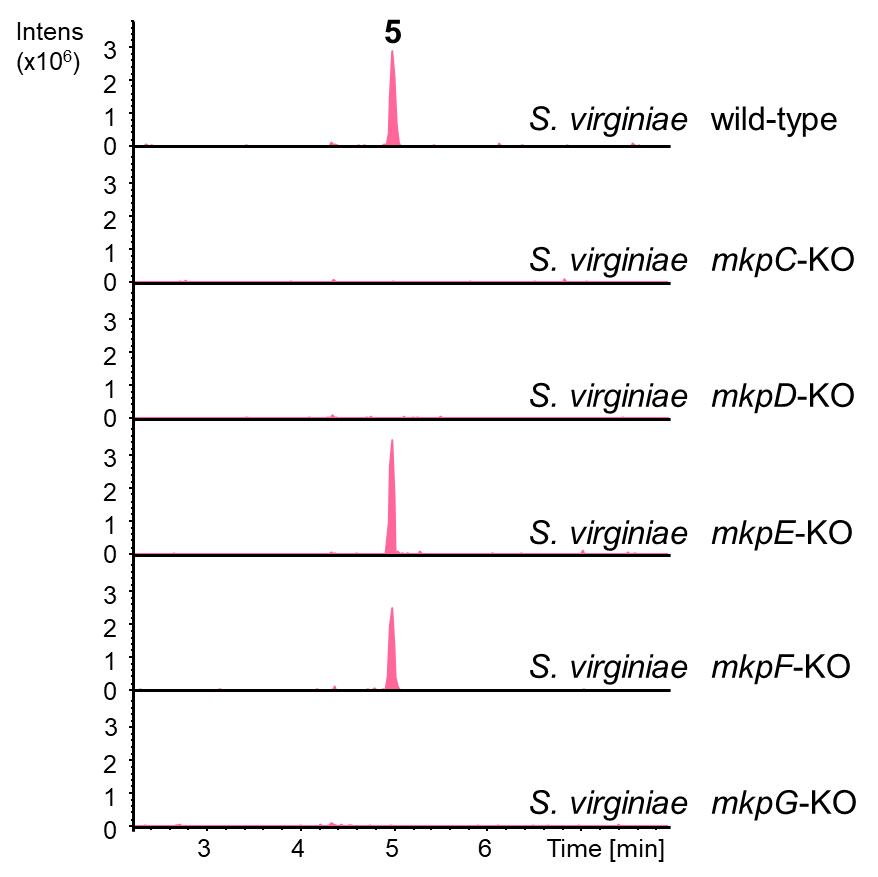
**

**Supplementary Figure 6** UPLC-MS analysis of *mkp* gene deletion mutants.

EICs for **5** (*m*/*z* 202.14) are shown. Knockout for *mkpC, mkpD,* and *mkpG* abolished the production of **5**.

**
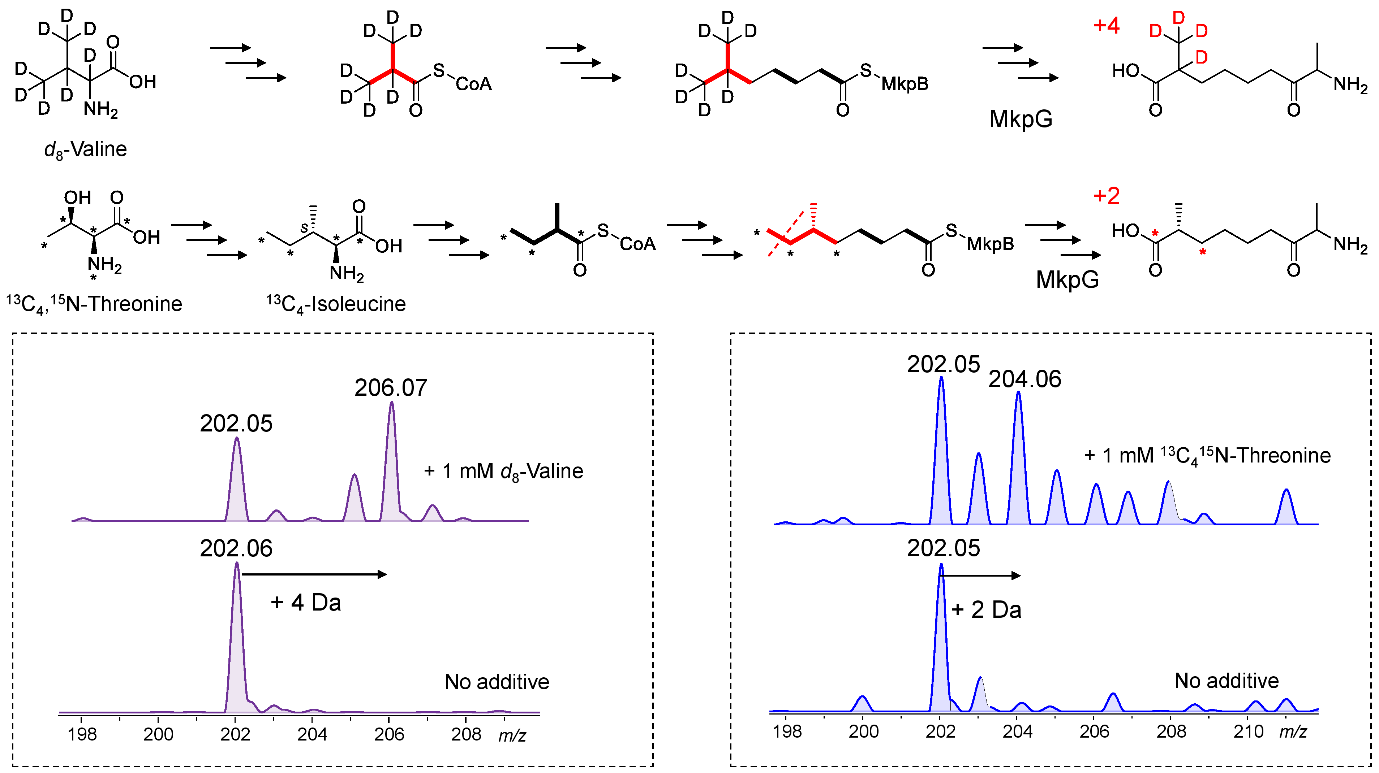
**

**c**

**b**

**a**


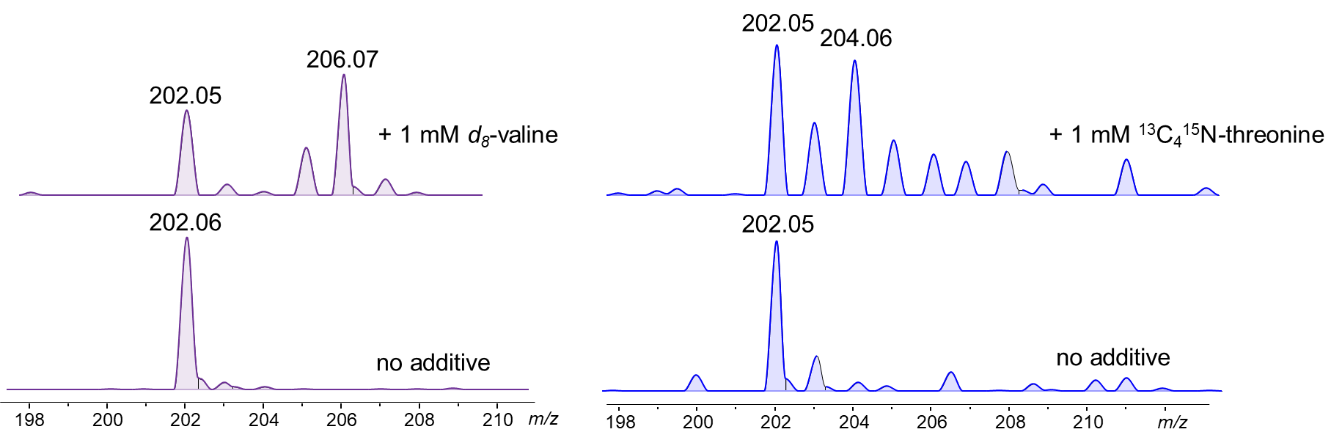


**Supplementary Figure 7** Isotope-labeling analysis for α-methyl-KAPA (**5**).

*S. virginiae* DSM40094 was employed for the isotope-labeling experiment. **a***,* The proposed labeling pattern upon feeding of *d8-*valine and ^13^C_4_,^15^N-threonine, resulting in +4 Da and +2 Da of mass shifts, respectively. **b***,***c**, The observed mass shifts for α-methyl-KAPA upon feeding of *d^8^-*valine (**b**) and ^13^C_4_,^15^N-threonine (**c**)*.*

**
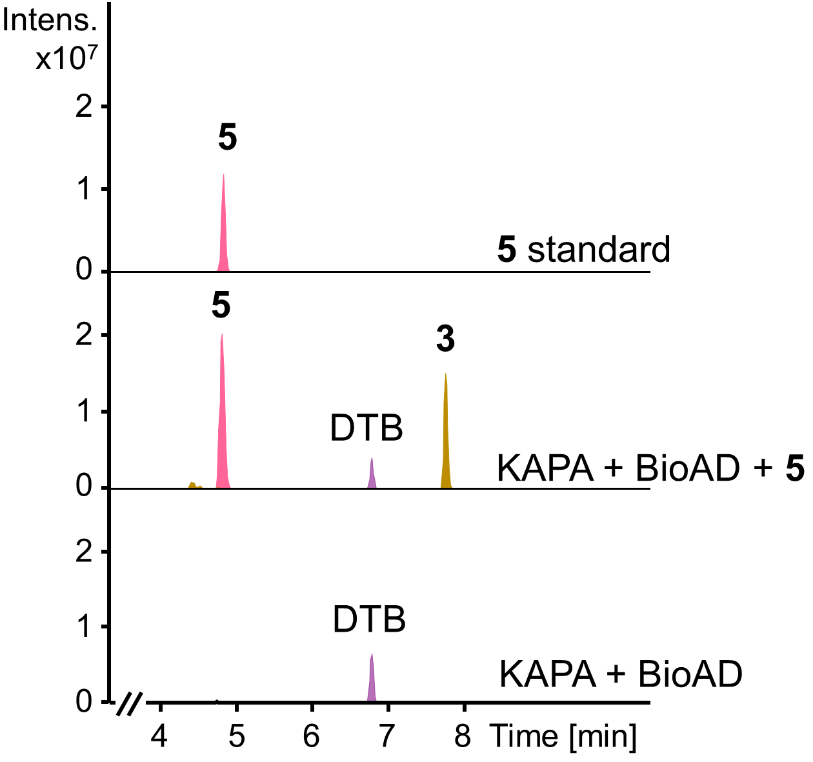
**

**Supplementary Figure 8** *In vitro* conversion of α-methyl-KAPA (**5**) to α-methyldesthiobiotin (**3**) by *E. coli* BioAD.

EICs for **3** (*m*/*z* 229.15), **5** (*m*/*z* 202.14), and desthiobiotin (DTB, *m*/*z* 215.14) are shown. **3** was produced by BioAD in the presence of **5** in addition to DTB.

**
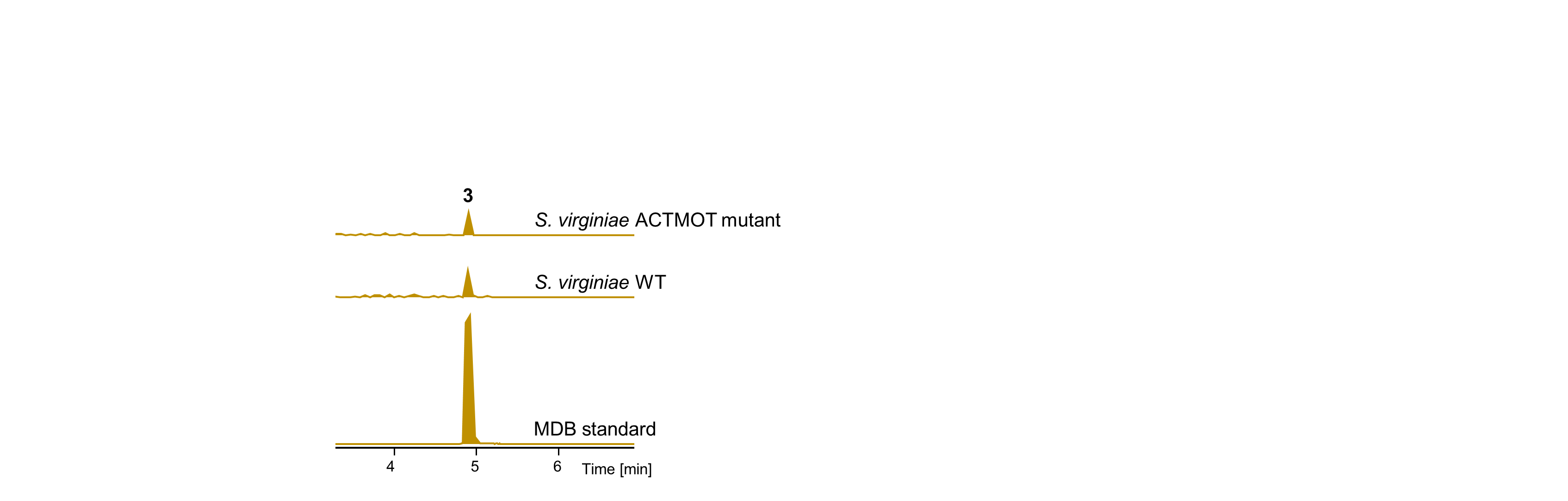
**

**Supplementary Figure 9** Detection of α-methyldesthiobiotin (**3**) in *S. virginiae* WT and its ACTIMOT mutant.

EICs for **3** (*m*/*z* 229.15) are shown. *S. virginiae,* which lacks additional copies of *bioA* and *bioD* present in *S. lydicus,* produced **3** but did not produce α-methylbiotin (**4**) (data not shown).

**
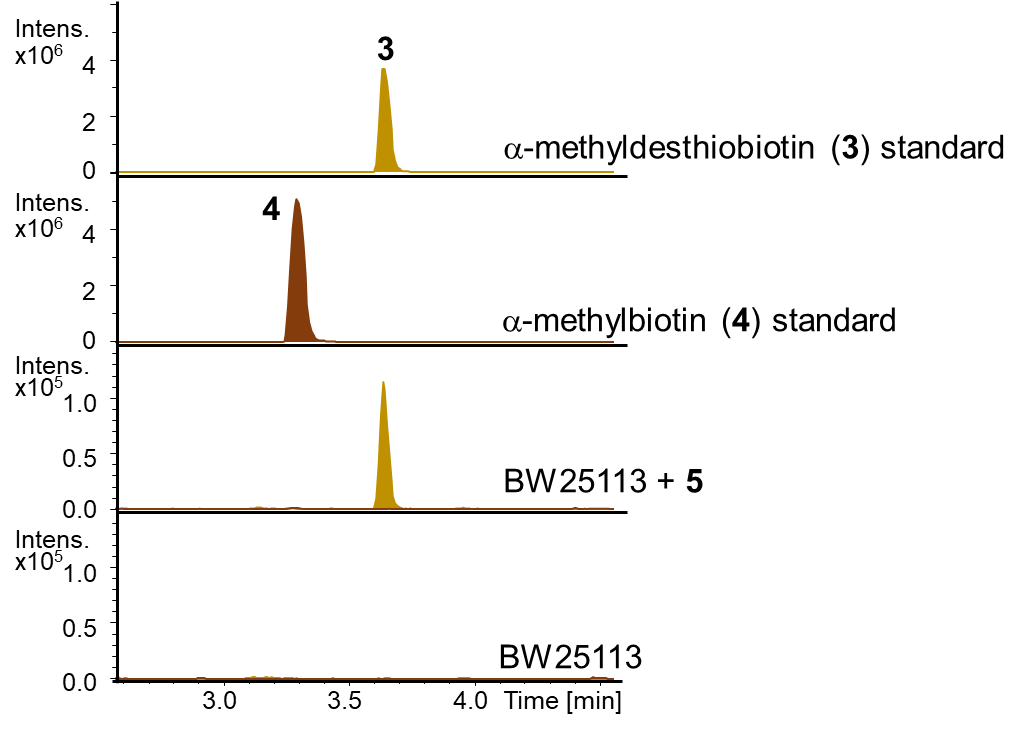
**

**Supplementary Figure 10** Detection of α-methyldesthiobiotin (**3**) in *E. coli* BW25113 treated with α-methyl-KAPA (**5**).

EICs for **3** (*m*/*z* 229.16) and **4** (*m*/*z* 259.11) are shown. Only α-methyldesthiobiotin (**3**), rather than α-methylbiotin (**4**), was detected in the crude extract. The samples were measured with ACQUITY UPLC BEH C18 column by a different method; the gradient changed from 5 to 95 % acetonitrile in 9 min instead of 18 min (See Methods “UPLC-MS analysis”). We assumed that methylated intermediate can interfere the reactions of BioA and BioD as a competitive inhibitor in both *Streptomyces* (Supplementary Figure 9) and *E. coli.*


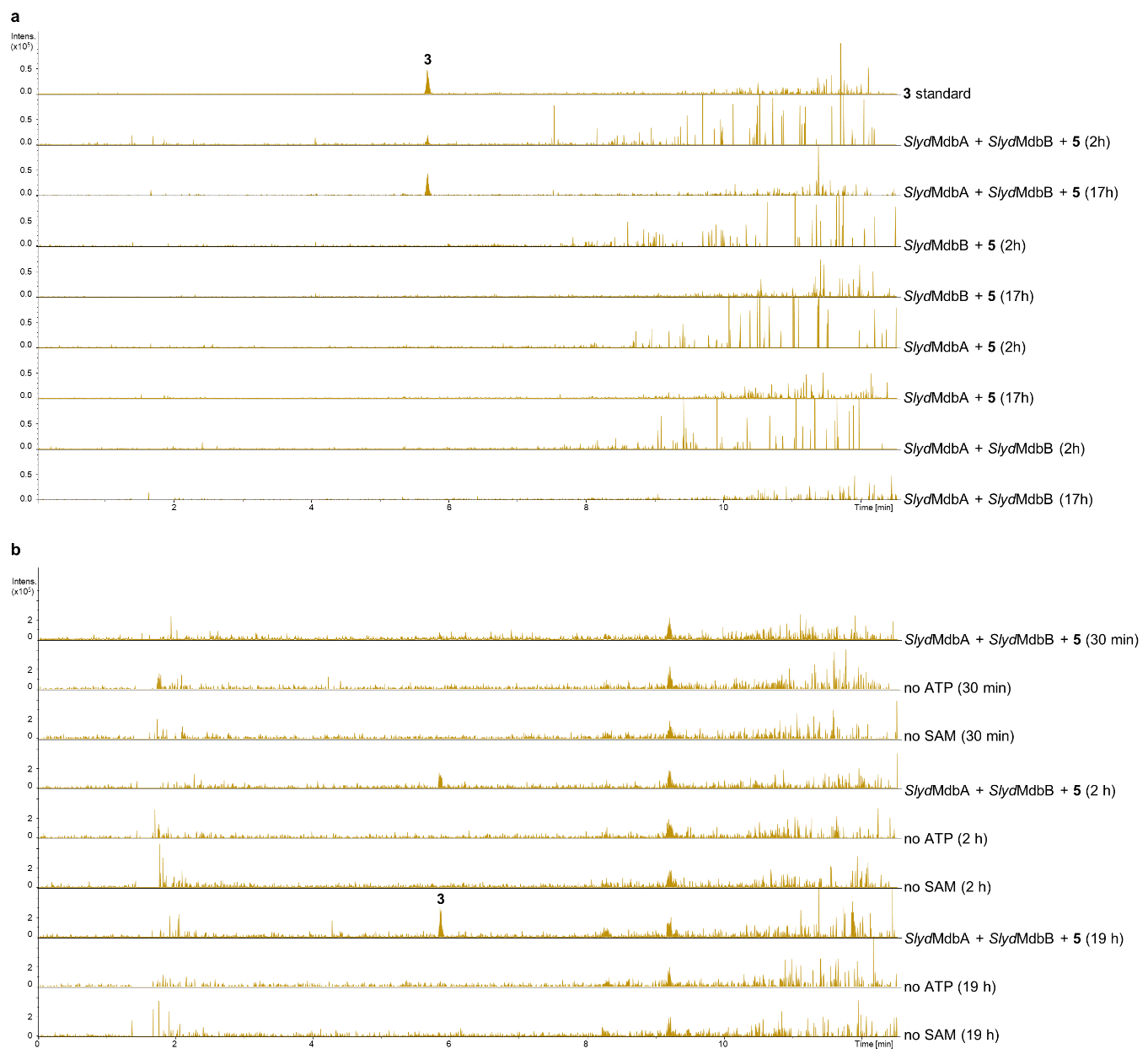


**Supplementary Figure 11** *In vitro* production of α-methyldesthiobiotin (**3)** from α-methyl-KAPA (**5**) using MdbA and MdbB.

**a**, *In vitro* assay with MdbA (14 µM) and MdbB (25 µM) derived from *S. lydicus*. EICs for **3** (*m*/*z* 211.15 [M^+^+H−H_2_O]) are shown. **3** was produced in a manner dependent on MdbA, MdbB and 5. The production was increased over a reaction time ranging from two hours to 17 hours. **b**, In vitro assay with MdbA (10 µM) and MdbB (10 µM) derived from *S. lydicus*. EICs for **3** (*m*/*z* 211.15 [M^+^+H−H_2_O]) are shown. **3** was produced in an ATP and SAM-dependent manner.

**
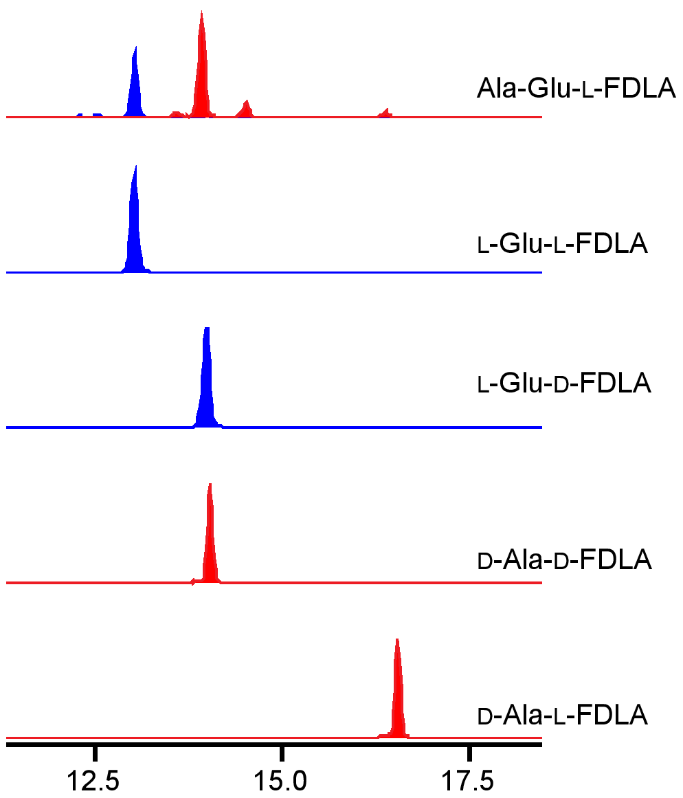
**

**Supplementary Figure 12** Marfey analysis of Ala-Glu dipeptide derived from Ala-ANDA (**10**).

EICs for Glu-FDLA (*m*/*z* 442.16) and Ala-FDLA (*m*/*z* 384.15). Both residues were determined to be l-configuration.

**
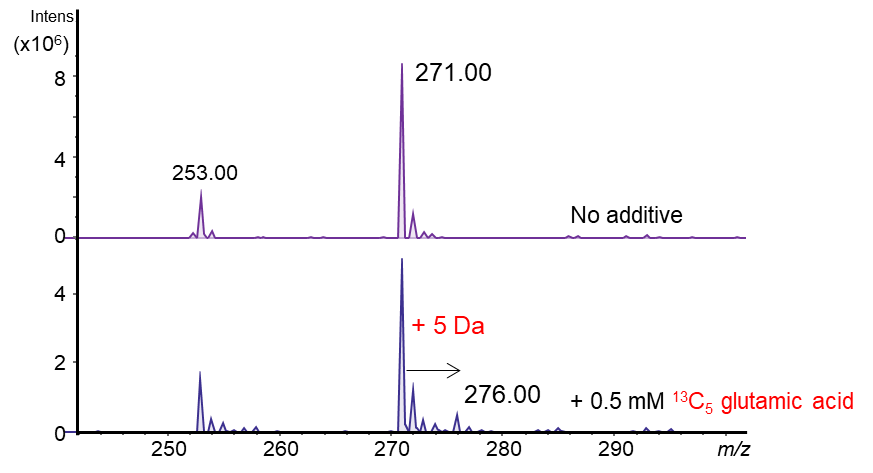
**

**Supplementary Figure 13** Isotope-labeling analysis for Ala-ANDA (**10**).

*S. albus* Del14:: p15A-*kasO*p*-*nda* was employed for the isotope-labeling experiment. The feeding of l-glutamic acid-^13^C_5_ led to a + 5 Da mass shift in Ala-ANDA.

**
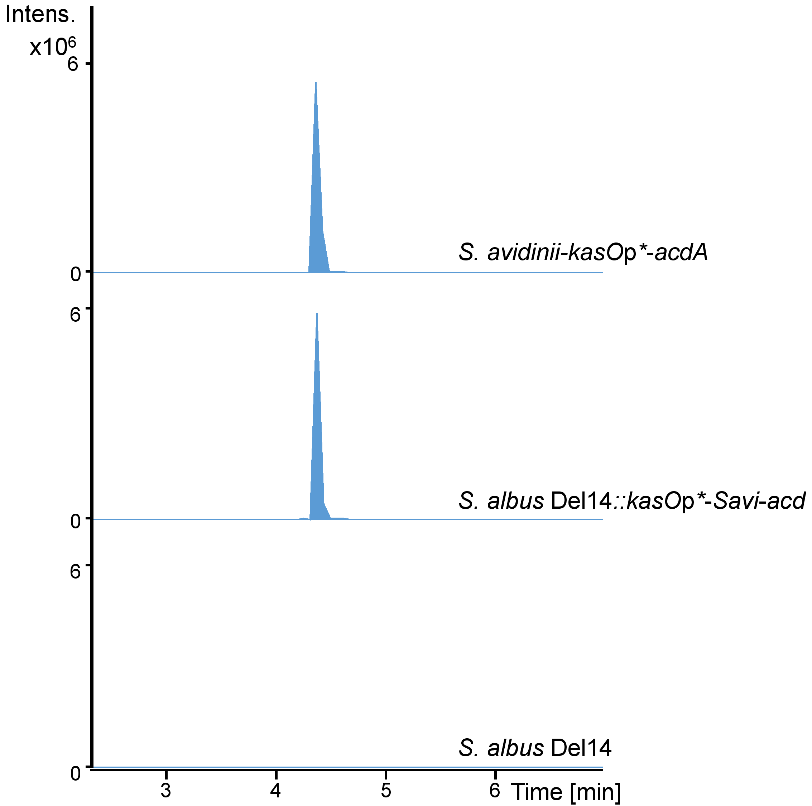
**

**1**

**Supplementary Figure 14** Production of acidomycin (**1**) in *S. avidinii*-*kasO*p*-*acdA* and *S. albus* Del14::*kasO*p*-*Savi*-*acd*.

EICs for **1** (*m*/*z* 218.08) are shown.

**
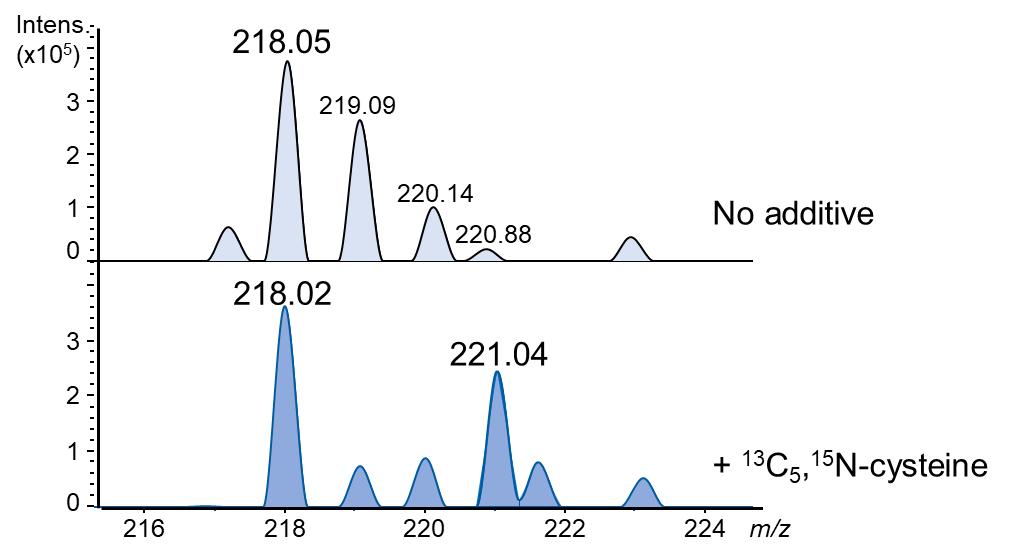
**

**Supplementary Figure 15** Isotope-labeling analysis for acidomycin (**1**).

The expected mass shift (Figure 4e) was observed upon feeding ^13^C_5_,^15^N-cysteine into the culture of *S. avidinii*-*kasO*p*-*acdA*.

**
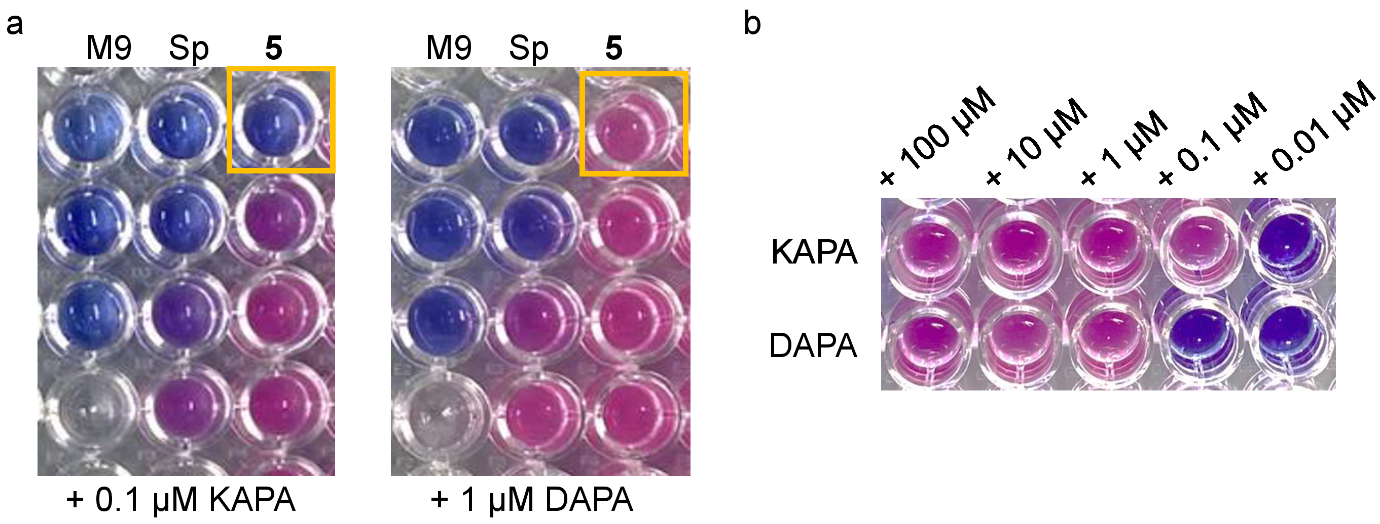
**

**Supplementary Figure 16** Rescue assay for α-methyl-KAPA (**5**).

The Keio mutant Δ*bioF* was employed. **a**, From the top row to the bottom row spectinomycin (Sp, MIC = 32 µM^1^) and **5** were serially diluted from 64 to 8 µM and 2.1 to 0.26 µM at the final concentration, respectively. After the incubation of the plate at 37 °C for 19 hours, resazurine was added to evaluate the viability. 0.1 µM KAPA did not rescue the growth of Δ*bioF,* while 1 µM DAPA rescued it as circled in yellow. **b**, The preliminary experiment to test the necessary amount of KAPA and DAPA to rescue the growth of Δ*bioF.* The strain required 0.1 µM KAPA and 1 µM DAPA at the final concentrations.


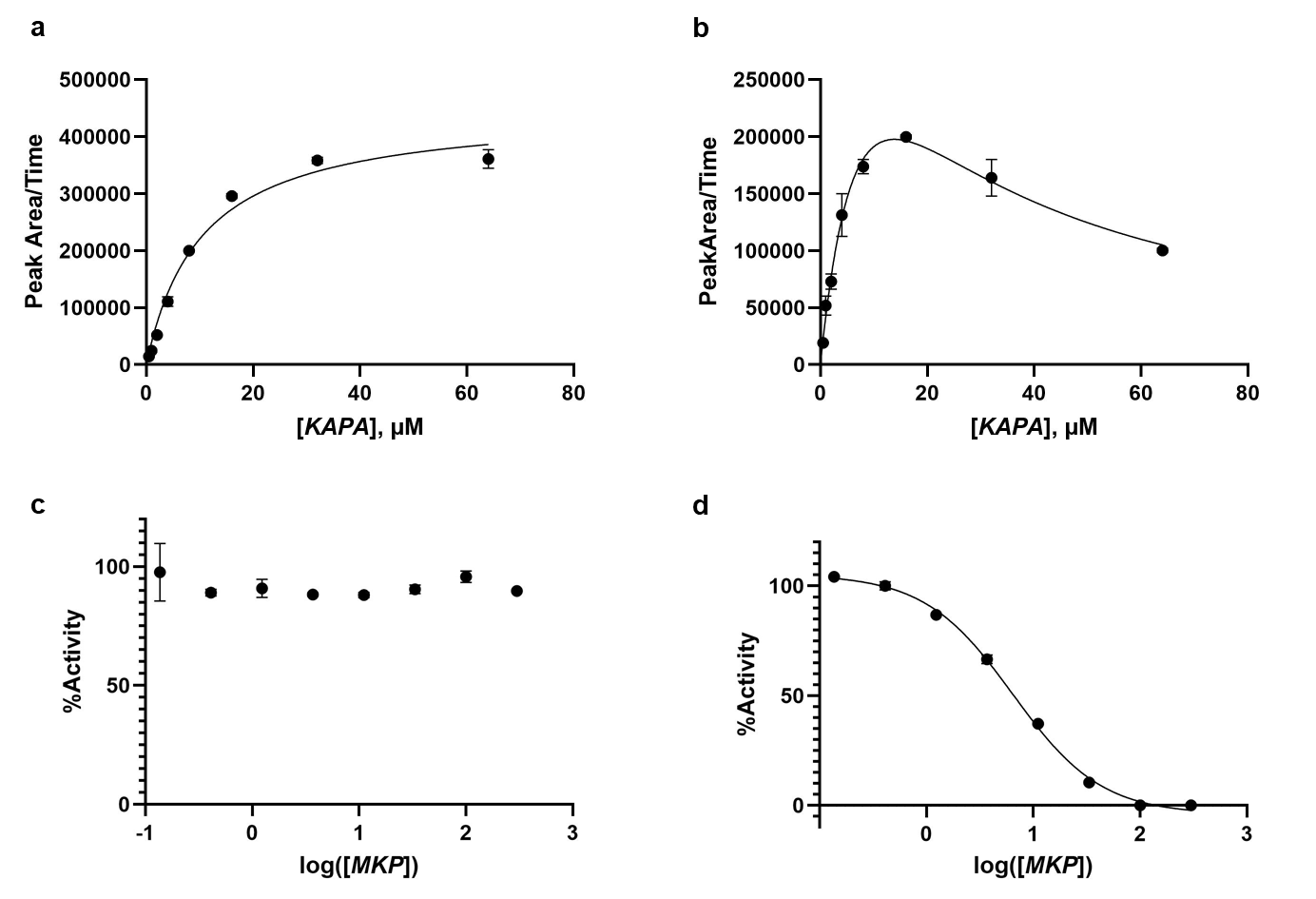


**Supplementary Figure 17** *In vitro* inhibition assay of BioAD with α-methyl-KAPA (**5**).

**a**,**b**, *Km* measurement for BioA derived from *E. coli* (**a**, *Ec*BioA) and *S. lydicus* (**b**, *Sl*BioA). The *Km* was determined as 10.35 µM and 11.40 µM by the curve-fit to Michaelis-Menten equation (*R^2^*=0.98) and Substrate inhibition model (*R^2^*=0.98), respectively. The *Ki*(KAPA) for *Sl*BioA was calculated as 16.84 µM in GraphPad. The KAPA concentration for the IC_50_ measurement was set to 10.35 µM equally to the *Km* value for *Ec*BioA and 13.86 µM for *Sl*BioA as calculated by the square of *Km·Ki*(KAPA)^2^. **c**, Bypass assay for BioAD coupling assay using 1.68 µM of DAPA as a substrate. The dose-response relationship was not observed, indicating that **5** is a competitive inhibitor for BioA rather than BioD. **d**, Dose-response relationship between **5** and BioA derived from *S. lydicus* (*R^2^*=0.99). *Ki(MKP)* was determined as 2.0 µM (See the Method for the equation). MKP: α-methyl-KAPA


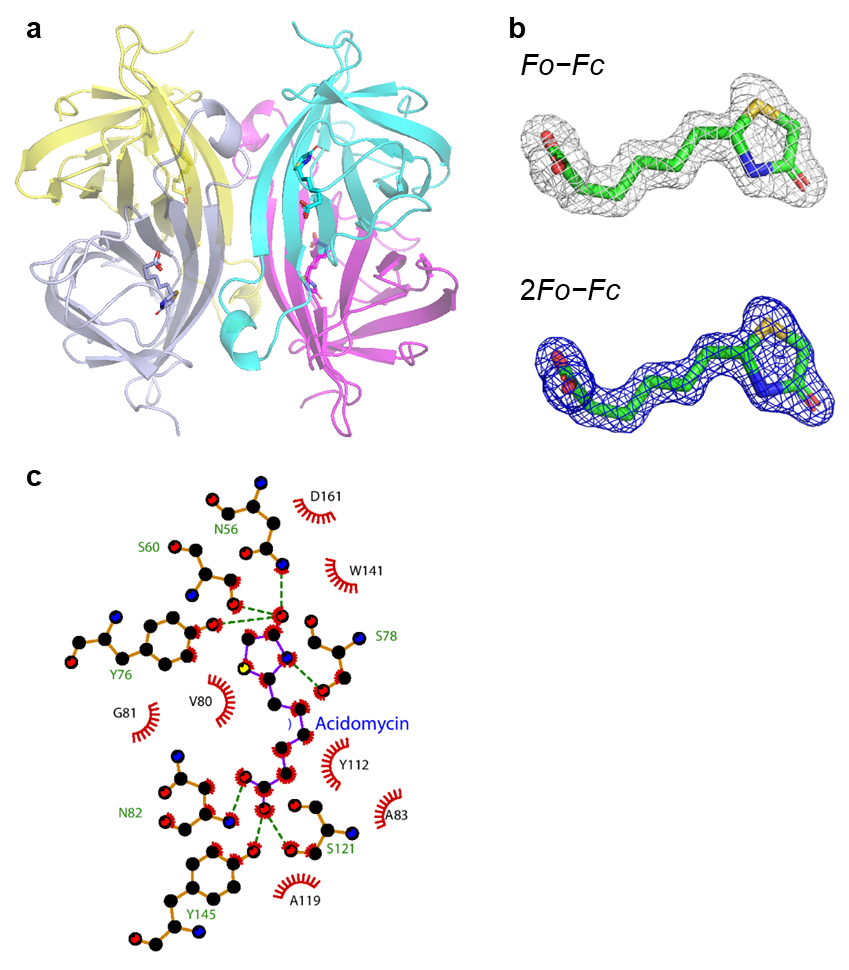


**Supplementary Figure 18** Structural analysis for streptavidin 2-acidomycin complex.

**a**, Overall crystal structure of the tetrameric streptavidin 2 (Sav2)-acidomycin complex through crystallographic symmetry operations. The structures of acidomycin are shown in stick. **b**, The *Fo* − *Fc* maps (contour level = 3.0 σ) and the 2 *Fo* − *Fc* omit maps (contour level = 1.0 σ) are shown as the grey and blue mesh, respectively, from the top to the bottom. **c**, Interactions between acidomycin and surrounding Sav2 residues, analyzed by the Ligplus+ program^3^.

## **Supplementary Figure 19** COSY and Key HMBC correlations of **8**.

## **Supplementary Figure 20** COSY and Key HMBC correlations of α-methyl-KAPA (**5**).

## **Supplementary Figure 21** COSY and Key HMBC correlations of **9**.

## **Supplementary Figure 22** COSY and Key HMBC correlations of α-methyldesthiobiotin (**3**).

## **Supplementary Figure 23** COSY and Key HMBC correlations of ANDA (**6**).

## **Supplementary Figure 24** COSY and Key HMBC correlations of Ala-ANDA (**10**).

## **Supplementary Figure 25** COSY and Key HMBC correlations of (*E*)-**6***.

## **Supplementary Figure 26** COSY and Key HMBC correlations of (*Z*)-**6***.

## **Supplementary Figure 27** Conversion of Ala-Glu from Ala-ANDA (**10**).

## **Supplementary Figure 28** COSY and Key HMBC correlations of acidomycin (**1**).

**NMR Figure**

**Supplementary Figure 29** ^1^H NMR spectrum of **8** (500 MHz, D_2_O).

**Supplementary Figure 30** HSQC spectrum of **8** (500 MHz, D_2_O).

**Supplementary Figure 31** HMBC spectrum of **8** (500 MHz, D_2_O).

**Supplementary Figure 32** COSY spectrum of **8** (500 MHz, D_2_O).

## **Supplementary Figure 33** ^1^H NMR spectrum of α-methyl-KAPA (**5**) (500 MHz, D_2_O).

## **Supplementary Figure 34** ^13^C NMR spectrum of α-methyl-KAPA (**5**) (125 MHz, D_2_O).

## **Supplementary Figure 35** HSQC spectrum of α-methyl-KAPA (**5**) (500 MHz, D_2_O).

## **Supplementary Figure 36** HMBC spectrum of α-methyl-KAPA (**5**) (500 MHz, D_2_O).

## **Supplementary Figure 37** COSY spectrum of α-methyl-KAPA (**5**) (500 MHz, D_2_O).

## **Supplementary Figure 38** ^1^H NMR spectrum of **9** (500 MHz, CD_3_OD).

## **Supplementary Figure 39** ^13^C NMR spectrum of **9** (125 MHz, CD_3_OD).

## **Supplementary Figure 40** HSQC spectrum of **9** (500 MHz, CD_3_OD).

## **Supplementary Figure 41** HMBC spectrum of **9** (500 MHz, CD_3_OD).

## **Supplementary Figure 42** COSY spectrum of **9** (500 MHz, CD_3_OD).

## **Supplementary Figure 43** ^1^H NMR spectrum of α-methyldesthiobiotin (**3**) (500 MHz, D_2_O).

## **Supplementary Figure 44** ^13^C NMR spectrum of α-methyldesthiobiotin (**3**) (125 MHz, D_2_O).

## **Supplementary Figure 45** HSQC spectrum of α-methyldesthiobiotin (**3**) (500 MHz, D_2_O).

## **Supplementary Figure 46** HMBC spectrum of α-methyldesthiobiotin (**3**) (500 MHz, D_2_O).

## **Supplementary Figure 47** COSY spectrum of α-methyldesthiobiotin (**3**) (500 MHz, D_2_O).

## **Supplementary Figure 48** ^1^H NMR spectrum of ANDA (**6**) (500 MHz, D_2_O).

## **Supplementary Figure 49** HSQC spectrum of ANDA (**6**) (500 MHz, D_2_O).

## **Supplementary Figure 50** HMBC spectrum of ANDA (**6**) (500 MHz, D_2_O).

## **Supplementary Figure 51** COSY spectrum of ANDA (**6**) (500 MHz, D_2_O).

## **Supplementary Figure 52** ^1^H NMR spectrum of Ala-ANDA (**10**) (500 MHz, CD_3_OD).

## **Supplementary Figure 53** ^13^C NMR spectrum of Ala-ANDA (**10**) (125 MHz, CD_3_OD).

## **Supplementary Figure 54** HSQC spectrum of Ala-ANDA (**10**) (500 MHz, CD_3_OD).

## **Supplementary Figure 55** HMBC spectrum of Ala-ANDA (**10**) (500 MHz, CD_3_OD).

## **Supplementary Figure 56** COSY spectrum of Ala-ANDA (**10**) (500 MHz, CD_3_OD).

## **Supplementary Figure 57** ^1^H NMR spectrum of (*E*)-**6*** (500 MHz, D_2_O).

## **Supplementary Figure 58** ^13^C NMR spectrum of (*E*)-**6*** (125 MHz, D_2_O).

## **Supplementary Figure 59** HSQC spectrum of (*E*)-**6*** (500 MHz, D_2_O).

## **Supplementary Figure 60** HMBC spectrum of (*E*)-**6*** (500 MHz, D_2_O).

## **Supplementary Figure 61** COSY spectrum of (*E*)-**6*** (500 MHz, D_2_O).

## **Supplementary Figure 62** ^1^H NMR spectrum of (*Z*)-**6*** (500 MHz, D_2_O).

## **Supplementary Figure 63** ^13^C NMR spectrum of (*Z*)-**6*** (125 MHz, D_2_O).

## **Supplementary Figure 64** HSQC spectrum of (*Z*)-**6*** (500 MHz, D_2_O).

## **Supplementary Figure 65** HMBC spectrum of (*Z*)-**6*** (500 MHz, D_2_O).

## **Supplementary Figure 66** COSY spectrum of (*Z*)-**6*** (500 MHz, D_2_O).

## **Supplementary Figure 67** ^1^H NMR spectrum of acidomycin (**1**) (500 MHz, CD_3_OD).

## **Supplementary Figure 68** ^13^C NMR spectrum of acidomycin (**1**) (125 MHz, CD_3_OD).

## **Supplementary Figure 69** HSQC spectrum of acidomycin (**1**) (500 MHz, CD_3_OD).

## **Supplementary Figure 70** HMBC spectrum of acidomycin (**1**) (500 MHz, CD_3_OD).

## **Supplementary Figure 71** COSY spectrum of acidomycin (**1**) (500 MHz, CD_3_OD).

Supplementary Tables

## **Supplementary Table 1.** Bacterial strains used in this work.

| **Strain** | **Description** | **Source or reference** |
| --- | --- | --- |
| *Escherichia coli* strains | | |
| DH10B | Host for cosmids and molecular cloning | Thermo |
| SCS110 | Donor strain for triparental conjugation, methylation deficient | Agilent |
| HB101/pRK2013 | Helper strain for triparental conjugation, Km^R^ | ^4^ |
| ET12567/pUZ8002 | Donor strain for biparental conjugation, methylation deficient, Chl^R^, Km^R^ | ^5^ |
| GB08Red | Tool strain for Red/ET recombineering | ^6^ |
| BL21 (DE3) | Protein expression host | Novagen |
| DH10B/p15A-cm-tetR-tetO-hyg-ccdB | DH10B harboring p15A-cm-tetR-tetO-hyg-ccdB | ^7^ |
| DH10B/pCap-SG5-Apr-SVI7-1-LR | DH10B harboring pCap-SG5-Apr-SVI7-1-LR | This study |
| DH10B/pRel-SVI7-1-dsp | DH10B harboring pRel-SVI7-1-dsp | This study |
| DH10B/pRelCap-SVI7-1-dsp | DH10B harboring pRelCap-SVI7-1-dsp | This study |
| ET12567/pUZ8002/pRelCap-SVI7-1-dsp | Used for conjugation | This study |
| DH10B/pCap-*Svi7-1* | DH10B harboring pCap-*Svi7-1* | This study |
| DH10B/p15A-int | DH10B harboring p15A-int | This study |
| DH10B/p15A-*kasO*p*-*mkp* | DH10B harboring p15A-*kasO*p*-*mkp* | This study |
| DH10B/p15A-*kasO*p*-*nda* | DH10B harboring p15A-*kasO*p*-*nda* | This study |
| DH10B/p15A-*kasO*p*-*Savi-acd* | DH10B harboring p15A-*kasO*p*-*acd* | This study |
| DH10B/p15A-cm-*mkp* | DH10B harboring p15A-cm-*mkp* | This study |
| DH10B/p15A-cm-*mkp*-*mkpC*KO | DH10B harboring p15A-cm-*mkp*-*mkpC*KO | This study |
| DH10B/p15A-cm-*mkp*-*mkpD*KO | DH10B harboring p15A-cm-*mkp*-*mkpD*KO | This study |
| DH10B/p15A-cm-*mkp*-*mkpE*KO | DH10B harboring p15A-cm-*mkp*-*mkpE*KO | This study |
| DH10B/p15A-cm-*mkp*-*mkpF*KO | DH10B harboring p15A-cm-*mkp*-*mkpF*KO | This study |
| DH10B/p15A-cm-*mkp*-*mkpG*KO | DH10B harboring p15A-cm-*mkp*-*mkpG*KO | This study |
| DH10B/p15A-*mdb* | DH10B harboring the p15A-*mdb* | This study |
| DH10B/p15A-*gapdh*p-*mdb* | DH10B harboring the p15A-*gapdh*p-*mdb* | This study |
| DH10B/p15A-*gapdh*p-*mdb*-*mdbA*KO | DH10B harboring the p15A-*gapdh*p-*mdb*-*mdbA*KO | This study |
| DH10B/p15A-*gapdh*p-*mdb*-*mdbB*KO | DH10B harboring the p15A-*gapdh*p-*mdb*-*mdbB*KO | This study |
| ET12567/pUZ8002/p15A-cm-*mkp*-*mkpC*KO | Used for conjugation | This study |
| ET12567/pUZ8002/p15A-cm-*mkp*-*mkpD*KO | Used for conjugation | This study |
| ET12567/pUZ8002/p15A-cm-*mkp*-*mkpE*KO | Used for conjugation | This study |
| ET12567/pUZ8002/p15A-cm-*mkp*-*mkpF*KO | Used for conjugation | This study |
| ET12567/pUZ8002/p15A-cm-*mkp*-*mkpG*KO | Used for conjugation | This study |
| ET12567/pUZ8002/p15A-*gapdh*p-*mdb* | Used for conjugation | This study |
| ET12567/pUZ8002/p15A-*gapdh*p-*mdb*-*mdbA*KO | Used for conjugation | This study |
| ET12567/pUZ8002/p15A-*gapdh*p-*mdb*-*mdbB*KO | Used for conjugation | This study |
| DH10B/pHistev | DH10B harboring pHistev | ^8^ |
| DH10B/pHisTev-*sav1* | DH10B harboring pHisTev-*sav1* | This study |
| DH10B/pHisTev-*sav2* | DH10B harboring pHisTev-*sav2* | This study |
| DH10B/pET28-*sav2* | DH10B harboring pET28-*sav2* | This study |
| BL21/pHisTev-*sav1* | BL21 (DE3) harboring pHisTev-*sav1* | This study |
| BL21/pHisTev-*sav2* | BL21 (DE3) harboring pHisTev-*sav2* | This study |
| BL21/pET28-*sav2* | BL21 (DE3) harboring pET28-*sav2* | This study |
| DH10B/pColdI-*SlydmdbA* | DH10B harboring pColdI-*SlydmdbA* | This study |
| DH10B/pColdI-*SlydmdbB* | DH10B harboring pColdI-*SlydmdbB* | This study |
| BL21/pColdI-*SlydmdbA* | BL21 (DE3) harboring pColdI-*SlydmdbA* | This study |
| BL21/pColdI-*SlydmdbB* | BL21 (DE3) harboring pColdI-*SlydmdbB* | This study |
| DH10B/pET28-*bioA* | DH10B harboring pET28-*bioA* | This study |
| DH10B/pET28-*bioD* | DH10B harboring pET28-*bioD* | This study |
| BL21/pET28-*bioA* | BL21 (DE3) harboring pET28-*bioA* | This study |
| BL21/pET28-*bioD* | BL21 (DE3) harboring pET28-*bioD* | This study |
| GB05RedTrfA | Tool strain for direct cloning | ^7^ |
| DH10B/SuperCos-A24 | DH10B harboring SuperCos-A24 | This study |
| DH10B/SuperCos-A24-int | DH10B harboring SuperCos-A24-int | This study |
| DH10B/SuperCos-A24-int-mini | DH10B harboring SuperCos-A24-int-mini | This study |
| DH10B/SuperCos-*kasO*p*-*Savi*-*acd* | DH10B harboring SuperCos-*kasO*p*-*Savi*-*acd* | This study |
| *Streptomyces* strains | | |
| *S. coelicolor* M145 | *S. coelicolor* plasmid-free strain | ^5^ |
| *S. virginiae* DSM 40094 | Origin of *Svi7-1* | DSM 40094 |
| *S. avidinii* DSM 40526 | Stravidins and streptavidin producer | DSM 40526 |
| *S. albus* Del14 | Host for heterologous expression | ^9^ |
| *S. virginiae*/pRelCap-SVI7-1-dsp  (*S. virginiae*/pCap-*Svi7-1*) | *S. virginiae* DSM 40094 conjugated with pRelCap-SVI7-1-dsp (*Svi7-1* mobilization exconjugant) | This study |
| *S. albus* Del14/pCap-*Svi7-1* | *S. albus* Del14 harboring pCap-*Svi7-1*, for heterologous expression of *Svi7-1* | This study |
| *S. albus* Del14::p15A-*kasO*p*-*mkp* | *S. albus* Del14 harboring BGC *mkp* (from *S. virginiae*) under the control of *kasO*p* | This study |
| *S. albus* Del14::p15A-*kasO*p*-*nda* | *S. albus* Del14 harboring BGC *nda* (from *S. virginiae*) under the control of *kasO*p* | This study |
| *S. albus* Del14::p15A*-kasO*p*-*Svir-acd* | *S. albus* Del14 harboring BGC *acd* (from *S. virginiae*) under the control of *kasO*p* | This study |
| *S. avidinii-kasO*p***-*acdA* | *S. avidinii* harboring BGC *acd* under promoter *kasO*p* | This study |
| *S. albus* Del14::*kasO*p***-*Savi*-*acd* | *S. albus* Del14 harboring BGC *acd* under the control of *kasO*p* | This study |
| *S. virginiae*-*mkpC*KO | *mkpC in situ* knock out mutant | This study |
| *S. virginiae*-*mkpD*KO | *mkpD in situ* knock out mutant | This study |
| *S. virginiae*-*mkpE*KO | *mkpE in situ* knock out mutant | This study |
| *S. virginiae*-*mkpF*KO | *mkpF in situ* knock out mutant | This study |
| *S. virginiae*-*mkpG*KO | *mkpG in situ* knock out mutant | This study |
| *S. lydicus* DSM 40461 | *S. lydicus* plasmid-free strain | ^10^ |
| *S. coelicolor*::p15A-*mdb* | *S. coelicolor* harboring the p15A-*mdb* | This study |
| *S. coelicolor*::p15A-*gapdh*p-*mdb* | *S. coelicolor* harboring the p15A-*gapdh*p-*mdb* | This study |
| *S. coelicolor*::p15A-*gapdh*p-*mdb*-*mdbA*KO | *S. coelicolor* harboring the p15A-*gapdh*p-*mdb*-*mdbA*KO | This study |
| *S. coelicolor*::p15A-*gapdh*p-*mdb*-*mdbB*KO | *S. coelicolor* harboring the p15A-*gapdh*p-*mdb*-*mdbB*KO | This study |

**Supplementary Table 2.** Plasmids used in this work.

| **Plasmid** | **Description** | **Source or reference** |
| --- | --- | --- |
| Plasmids for BGC mobilization | | |
| pCap-SG5-Apr | Capture plasmid, Apramycin resistant, pSG5 replicon | ^11^ |
| pRel | Release plasmid, Apramycin resistant, pSG5 replicon | ^11^ |
| pCap-SG5-Apr-SVI7-1-LR | pCap for ACTIMOT application of the *Svi7-1* | This study |
| pRel-SVI7-1-dsp | pRel for ACTIMOT application of the *Svi7-1* | This study |
| pRelCap-SVI7-1-dsp | Working plasmid for ACTIMOT application of the *Svi7-1* | This study |
| Plasmids for BGCs heterologous expression | | |
| pCap-*Svi7-1* | Plasmid harboring *Svi7-1*, Apr^R^ | This study |
| p15A-int | Modified p15A vector with φC31 integrase cassette, Apr^R^ | This study |
| p15A-int-*kasO*p*-*sf*p-amp | Modified p15A-int vector with *Streptomyces* promoters pair *kasO*p* and sfp Apr^R^, Amp^R^ | This study |
| p15A-*kasO*p*-*mkp* | BGC *mkp* heterologous expression vector | This study |
| p15A-*kasO*p*-*nda* | BGC *nda* heterologous expression vector | This study |
| p15A-*kasO*p*-*Savi-acd* | BGC *acd* heterologous expression vector | This study |
| SuperCos 1 | Tool vector for cosmid library construction | Agilent |
| SuperCos-A24 | Cosmid harboring BGC *acd* | This study |
| SuperCos-A24-int | SuperCos-A24 with integrase gene | This study |
| SuperCos-A24-int-mini | Minimized SuperCos-A24-int, harboring BGC *acd* and one redundant gene | This study |
| SuperCos-*kasO*p**-Savi-acd* | SuperCos-A24-int-mini with a protomer *kasO*p* | This study |
| Plasmid for acidomycin production optimization | |  |
| pQS-gusA-*acdA*-*kasO*p* | Tool plasmid for introducing *kasOp* to BGC *acd* upstream region | This study |
| Plasmids for gene knock out | | |
| p15A-cm-tetR-tetO-hyg-ccdB | Tool plasmid for direct cloning | ^7^ |
| p15A-cm-*mkp* | p15A-cm-tetR-tetO-hyg-ccdB backbone, harboring *mkp* BGC | This study |
| p15A-cm-*mkp*-*mkpC*KO | *mkpC* gene knock out vector | This study |
| p15A-cm-*mkp*-*mkpD*KO | *mkpD* gene knock out vector | This study |
| p15A-cm-*mkp*-*mkpE*KO | *mkpE* gene knock out vector | This study |
| p15A-cm-*mkp*-*mkpF*KO | *mkpF* gene knock out vector | This study |
| p15A-cm-*mkp*-*mkpG*KO | *mkpG* gene knock out vector | This study |
| Plasmids for protein overexpression | | |
| pHisTev | Expression vector in *E. coli*, Kan^R^ | ^8^ |
| pET28b | Expression vector in *E. coli*, Kan^R^ | Novagen |
| pHisTev-*sav1* | Sav1 protein expression vector | This study |
| pHisTev-*sav2* | Sav2 protein expression vector | This study |
| pET28-*sav2* | Sav2 protein expression vector, for crystalization | This study |
| pET28-*bioA* | BioA expression vector | This study |
| pET28-*bioB* | BioD expression vector | This study |
| pColdI | Expression vector in *E. coli*, Amp^R^ | Takara |
| pColdI-*SlydmdbA* | *Slyd*MdbA protein expression vector | This study |
| pColdI-*SlydmdbB* | *Slyd*MdbB protein expression vector | This study |

## **Supplementary Table 3.** Primers used in this work.

| **Primers** | **Sequences** |
| --- | --- |
| For mobilization of *Svi7-1* | |
| Svi7-1-CapL-F | GATGGAGCTGCACATGAACCGGTCTTCTCGGCCGGGAGTT |
| Svi7-1-CapL-R | CACACAGTTTAAACGCAGCATGCCGAGTGAGGACCTAGTACTCACCGAACACGGAATGGTCGGCGCCGAGGTTGAT |
| Svi7-1-CapR-F | TGCTGCGTTTAAACTGTGTGAAATTGTTATCCGCCTGTGACCTTCTCTTCTTCAAGCTCCACCGAACTGCTCTCCT |
| Svi7-1-CapR-R | ATCAGACTATCAGCGTGAGAGTGGCAAGGTCACCGCGGAT |
| Svi7-1-sp1-F | CATGGTTCCGTGTTCGGTGAGTACTGTTTTAGAG |
| Svi7-1-sp1-R | CTAGCTCTAAAACAGTACTCACCGAACACGGAAC |
| Svi7-1-sp2-F | CATGGCTTGAAGAAGAGAAGGTCACGTTTTAGAG |
| Svi7-1-sp2-R | CTAGCTCTAAAACGTGACCTTCTCTTCTTCAAGC |
| Svi7-1-DelL-F | TCTCGTCGAAGGCACTAGAAGGGGCCACGGATCTCTCCTCGT |
| Svi7-1-DelL-R | ACCGCCAAGTCCGTGGTGCT |
| Svi7-1-DelR-F | AGCACCACGGACTTGGCGGTTCGCTCTCCAGCTTCGCCGT |
| Svi7-1-DelR-R | GCGGTCGATCCCCGCATATAGGGCCAAGCTGTCCCTGTGGAA |
| For BGCs heterologous expression | |
| p15A-F | CTCGAGAGATCCGAAAACCCCAAGTT |
| p15A-R | CTCGAGAGATCCTTTCTCCTCTTTAG |
| p15A-at-F | ATGGACACTCCTTACTTAGA |
| p15A-at-R | AACTCCCCCAGTCCTGCACG |
| p15a-mkp-1F | GAATTCAAAAGATCTAAAGAGGAGAAAGGATCTCTCGAGGTTCCACGTGTAGGAGTCAT |
| p15a-mkp-1R | AACAGGAAGCTGCGGGCCATGTTCATGACGTGGTCGACGA |
| p15a-mkp-2F | TCGTCGACCACGTCATGAACATGGCCCGCAGCTTCCTGTT |
| p15a-mkp-2R | CCTTAAGATCCGTAACTTGGGGTTTTCGGATCTCTCGAGACACCTTCACCAAGGTGAAG |
| mkp-kasop-F | CCCGCCGGTGCGGTGCGGGAGCCCGTGCCCGAAGTGTGATCAGTGGAACGAAAACTCAC |
| mkp-kasop-R | GCAGGTGGTGAGCCGGTCGAAAACGTCGTCGTTAGCCACATGGACACTCCTTACTTAGA |
| p15a-nda-1F | GAATTCAAAAGATCTAAAGAGGAGAAAGGATCTCTCGAGTGTTCACATTCGAACCGTCT |
| p15a-nda-1R | GTTCATCGCTGAGTGGTCCTCTTCTGGTCGTTCATGCGTC |
| p15a-nda-2F | GACGCATGAACGACCAGAAGAGGACCACTCAGCGATGAAC |
| p15a-nda-2R | AGATGGTGGGGAACTCGAAGAGCATGCCGATGCTCAGCTC |
| p15a-nda-3F | GAGCTGAGCATCGGCATGCTCTTCGAGTTCCCCACCATCT |
| p15a-nda-3R | CCTTAAGATCCGTAACTTGGGGTTTTCGGATCTCTCGAGTCATCGATACCGCTGGTCAT |
| nda-kasop-F | GCGCAGAGCGCGGACCGTCTGGTCGGGATGTAGGTCCATAACTCCCCCAGTCCTGCACG |
| nda-kasop-R | GAGCAGGCCGCCACGGCCGTGTCCGCCTGGCGCGTCTGAAATTTCTGCCATTCATCCGC |
| p15a-acd-vir-mini-1F | CGTGCAGGACTGGGGGAGTTGTGTTGCGCAAGATCTCTAC |
| p15a- acd-vir-mini-1R | AGACGCGTGAGGAGTAGTAGTCCAT |
| p15a acd-vir-mini-2F | TGGTCATGGACTACTACTCCTCACGCGTCTTCTACGAGGA |
| p15a-acd-vir-mini-2R | TCTAAGTAAGGAGTGTCCATATGACCAGCGGTATCGATGA |
| p15a-acdtrans-F | CACAGCGCTGAGTGCATATATCAACTCGGCGGCGGGGTGG |
| p15a-acdtrans-R | ACAAGGTTTTTCACTAGAGATGCGGAAATCCCCCTCGGTG |
| mdbAB-gb-F | AGAGGAGAAAGGATCTCTCGAGTTAATTAATACGAGCTGGTCCTGGAC |
| mdbAB-gb-R | GTTTTCCCTTCCCGTACGGACGTGTGCGAGCCGTTCTCGG |
| mdbCDF-gb-F | CCGAGAACGGCTCGCACACGTCCGTACGGGAAGGGAAAAC |
| mdbCDF-gb-R | CGGGCGCGGCAGCGCGCCGGAAGAACCGAGGGAGCTGTTG |
| mdbGH-gb-F | CAACAGCTCCCTCGGTTCTTCCGGCGCGCTGCCGCGCCCG |
| mdbGH-gb-R | GGGGTTTTCGGATCTCTCGAGGAAAAAGCGCAGGTCGGAG |
| mdb-gapdhp-Cm-F | AGCACTTCCACCGCGCACGACCGGACTGTCTCGAACACTGCATCGCTCATGCGTATCCCCTTTCAGATAC |
| mdb-gapdhp-Cm-R | TGCCTGGAGATCCTTAAGATCCGTAACTTGGGGTTTTCGGATCTCTCGAGAATTTCTGCCATTCATCCGCTT |
| Cos-screen-F1 | CGTTCATCATCGGTCCTTGT |
| Cos-screen -R1 | CAGCTCGAACTGAGCGTCAA |
| Cos-screen -F2 | TCATGCCCCACAAGGTGATT |
| Cos-screen -R2 | AGCAGCACCATGGACACCAC |
| Cos-integraseF | TCACCTAGATCCTTTTAAATTAAAAATGAAGTTTTAAATGGTTCATGTGCAGCTCCATC |
| Cos-integraseR | GCCTATTTTTATAGGTTAATGTCATGATAATAATGGTTTTATTTACCCGCAGGACATATCC |
| A24int-BspT1-Cm-F | TCTTCAAGAATTCGCGGCCGCAATTAACCCTCACTAAAGGCTTAAGAATTTCTGCCATTCATCCGC |
| Cm-BspT1-A24int-R | CGAACGCTCCGAGAGCGAGCGTCAAGACCTTGCCCAACACCTTAAGTGTTGATACCGGGAAGCCCT |
| A24int-staP-F | CAATTAACCCTCACTAAAGGCACGACCTCAGTACTGAATA |
| A24int-staP-F | CAATTAACCCTCACTAAAGGCACGACCTCAGTACTGAATA |
| For gene knock-out | |
| mkp-direct cloning-F | GGAGGTCCTCGTAGACCTCCAGGAACTGGTTCTGCGCACCGTCGTACAGATCCAGCAGCAGCACGACCCGAAGCCTCGAGAGATCCGAAAACCCCAAGTTACG |
| mkp-direct cloning-R | TCCACTACGGGCTCTGCGTCTTCGAGGGCATCTCCGCCTTCCCCTCGGCGGAGGGCGGCCACCACGTCTTCCGGCTCGAGAGATCCTTTCTCCTCTTTAGATC |
| mkpCKO-F | TCCAGACCGTCGGCGACCTGCACCGACTGATCCTGTCGCTGGTCGCCTGATGATTCCGGGGATCCGTCGA |
| mkpCKO-R | CGTCGTGCGGATCGCTGTGTCGCTCTGTTTCCATCGCTTCGCTCCGAACGCTATGTAGGCTGGAGCTGCT |
| mkpDKO-F | CGGCGTCCTCGTCCTCGGCCCTGCCGGATAGCGTTCGGAGCGAAGCGATGATTCCGGGGATCCGTCGACC |
| mkpDKO-R | CCTTCCGTCACTCGGTCCGGGCCGTCGTCCGCCCCGGGTCGGCTGCCCTATGTAGGCTGGAGCTGCTTCG |
| mkpEKO-F | GCCGACCCGGGGCGGACGACGGCCCGGACCGAGTGACGGAAGGCGTCATGATTCCGGGGATCCGTCGACC |
| mkpEKO-R | GATCGGGGTACGCCGCGCCGCCGCGACGATGGGCAGCTGCCCGTTCACAGTGTAGGCTGGAGCTGCTTCG |
| mkpFKO-F | CGCAGCGCCCTCCTGGAGGGTCGGCACGACATGCGGGAGGTTCTGTGAACATTCCGGGGATCCGTCGACC |
| mkpFKO-R | GGGGCTGACCCCGCCGCTCCCGCAGCGCGCCGTCCGGCCGGCCCGCGTCATGTAGGCTGGAGCTGCTTCG |
| mkpGKO-F | CCGACTGACGCGGGCCGGCCGGACGGCGCGCTGCGGGAGCGGCGGGGTCATGTAGGCTGGAGCTGCTTCG |
| mkpGKO-R | CGCTGGACCCGTATCCGGCGATGGCGGCGCTCCGAGAACACGAACCCGTGATTCCGGGGATCCGTCGACC |
| mdbA-KO-amp-F | CTGACCGGCCGGCTCCCGAGGCGCCATTCCGCGCGCCGCGCGACGCGCCTCGCGGAACCCCTATTTGT |
| mdbA-KO-amp-R | TCTCTCCCTCGTCTCGGCCCGATGCCCGCGTCGCTTCGTGCCCGTTGCCGTTACCAATGCTTAATCAGTGAGG |
| mdbB-KO-amp-F | GGCAACGGGCACGAAGCGACGCGGGCATCGGGCCGAGACGAGGGAGAGACCGCGGAACCCCTATTTGT |
| mdbB-KO-amp-R | CCGTGCCGGGAAAGGACGCAGTCCGGCCAGCGGTGTGCGGGGGCTTCCGCTTACCAATGCTTAATCAGTGAGG |
| For acidomycin production optimization | |
| acdAup-kasop-sgF | CATGGTTGGCCTGCAACTTCTGTTGGTTTTAGAG |
| acdAup-kasop-sgR | CATGGTTGGCCTGCAACTTCTGTTGGTTTTAGAG |
| acdAup-kasop-uF | CTCGTCGAAGGCACTAGAGGGGCGAGGAGATCCAGAACTC |
| acdAup-kasop-uR | GCCACGACTTTACAACACCGCACAGCATGTTGTCAAAGCAGAGACGGTTCGAATGTGAACACCTACGACCGGATCACTCCT |
| acdAup-kasop-dF | GCGGTGTTGTAAAGTCGTGGCCCGTGCAGGACTGGGGGAGTTGTGTTGGGCAAGGTCTTGAC |
| acdAup-kasop-dR | GGTCGATCCCCGCATATAGGGTAAGTCGGGGTGAGAGGGC |
| For protein expression | |
| Sav1-F | AAAACCTGTATTTTCAGGGCGCCATGGACCCCTCCAAGGACTCGAA |
| Sav1-R | TGGTGCTCGAGTGCGGCCGCAAGCTTCTACTGCTGAACGGCGTCGA |
| Sav2-F | AAAACCTGTATTTTCAGGGCGCCATGGACACCCCGGCGGCCGCGTC |
| Sav2-R | TGGTGCTCGAGTGCGGCCGCAAGCTTTCACTCCTGGCCGGAAGCCG |
| Sav2-pet-F | CCGCGCGGCAGCCATATGAACGGCCGCCCGTCCGTGCTC |
| Sav2-pet-R | TCCTTTCGGGCTTTGTTAGGAGGGCTTTACCCGGCTGAACTCGTC |
| bioA-28b-F | TGGTGCCGCGCGGCAGCCATACAACGGACGATCTTGCCTT |
| bioA-28b-R | TCGAGTGCGGCCGCAAGCTTTTATTGGCAAAAAAATGTTT |
| bioD-28b-F | TGGTGCCGCGCGGCAGCCATAGTAAACGTTATTTTGTCAC |
| bioD-28b-R | TCGAGTGCGGCCGCAAGCTTTTACAACAAGGCAAGGTTTA |
| *Slyd*MdbA-pCold-F | CATATCGAAGGTAGGCATATGCTCTCGGCCGTGACGCCT |
| *Slyd*MdbA-pCold-R | CAGGTCGACAAGCTTGAATTCTCAGCCGGCCCGTGCGGC |
| *Slyd*MdbB-pCold-F | CATATCGAAGGTAGGCATATGGCGCTGCTGGCCGTCACC |
| *Slyd*MdbB-pCold-R | CAGGTCGACAAGCTTGAATTCTCAGGAGTGCCACCGTGC |

## **Supplementary Table 4.** Gene annotation of the *sta*, *acd*, *mpk*, *acd*, and *mdb*.

| **Gene annotations of *sta* from *S. virginiae* and *S. avidinii*** | | |
| --- | --- | --- |
| **Gene** | **Annotation** | **Proposed function in the biosynthetic pathway** |
| *staA* | Aminodeoxychorismate synthase component I | Unknown function |
| *staB* | GNAT family *N*-acetyltransferase | Formation of the amiclenomycin residue |
| *staC* | Hypothetical protein | Unknown function |
| *staD* | Chorismate mutase | Formation of the amiclenomycin residue |
| *staE* | Hypothetical protein | Unknown function |
| *staF* | LeuA family protein | Formation of the amiclenomycin residue |
| *staG* | Class I SAM-dependent methyltransferase | Methylation of the *N*-terminal amino acid residue |
| *staH* | GNAT family *N*-acetyltransferase | Unknown function |
| *staI* | LeuD/DmdD family oxidoreductase small subunit | Formation of the amiclenomycin residue |
| *staJ* | Isocitrate/isopropylmalate dehydrogenase family protein | Formation of the amiclenomycin residue |
| *staK* | Aminotransferase class IV | Formation of the amiclenomycin residue |
| *staL* | Hypothetical protein | Unknown function |
| *staM* | MFS transporter | Transporter |
| *staN* | 3-deoxy-7-phosphoheptulonate synthase | Precursor synthesis |
| **Gene annotations of *acd* from *S. virginiae* and *S. avidinii*** | | |
| **Gene** | **Annotation** | **Proposed function in the biosynthetic pathway** |
| *acdA* | MFS transporter | Transporter |
| *acdB* | SDR family NAD(P)-dependent oxidoreductase | KR |
| *acdC* | Amino acid adenylation domain-containing protein | NRPS component |
| *acdD* | AMP-binding protein | Acyl-CoA ligase |
| *acdE* | Cytochrome P450 | Hydroxylation |
| *acdF* | 4'-phosphopantetheinyl transferase family protein | Activation of PCP dmains |
| **Gene annotations of *mkp* from *S. virginiae* and *S. avidinii*^1^** | | |
| **Gene** | **Annotation** | **Proposed function in the biosynthetic pathway** |
| *mkpH_avi_* | Adenosylmethionine-8-amino-7-oxononanoate transaminase | Unknown function |
| *mkpA* | MFS transporter | Transporter |
| *mkpB* | Acyl carrier protein | Formation of polyketide scaffold, ACP |
| *mkpC* | Beta-ketoacyl-acyl-carrier-protein synthase family protein | Formation of polyketide scaffold, KS |
| *mkpD* | 8-amino-7-oxononanoate synthase | Transfers amino group from l-alanine |
| *mkpE* | AMP-binding protein | Acyl-CoA ligase |
| *mkpF_vir_* | Thiolase family protein | Formation of the chemical scaffold |
| *mkpG* | Cytochrome P450 | Hydroxylation of polyketide intermediates |
| **Gene annotations of *nda* from *S. virginiae*** | | |
| **Gene** | **Annotation** | **Proposed function in the biosynthetic pathway** |
| *ndaA* | Nonribosomal peptide synthetase (NRPS) | NRPS modules incorporate activated amino acids into the polyketide moiety |
| *ndaB* | Type I polyketide synthase | Formation of the polyketide moiety |
| *ndaC* | Thioesterase II family protein | Releasing polyketide or hybrid product |
| *ndaD* | MFS transporter | Transporter |
| **Gene annotations of *mdb* from *S. lydicus*** | | |
| **Gene** | **Annotation** | **Proposed function in the biosynthetic pathway** |
| *mdbA* | BioA | BioA |
| *mdbB* | BioD | BioD |
| *mdbC* | BioB | BioB |
| *mdbD* | Cytochrome P450 | Cytochrome P450 |
| *mdbE* | 8-Amino-7-oxononanoate synthase | 8-Amino-7-oxononanoate synthase |
| *mdbF* | Beta-ketoacyl-acyl-carrier-protein synthase family protein | Beta-ketoacyl-acyl-carrier-protein synthase family protein |
| *mdbG* | Acyl carrier protein | Acyl carrier protein |

^1^ The specific genes in *mkp* from *S. virginiae* or *S. avidinii* are labeled with subscript *vir* or *avi*, respectively.

## **Supplementary Table 5.** Data collection and refinement statistics.

|  | Sav2-acidomycin |
| --- | --- |
| **PDB ID** | 9XIK |
| **Data collection** |  |
| Space group | *I*4_1_22 |
| Cell dimensions |  |
| *a*, *b*, *c* (Å) | 57.86, 57.86, 184.20 |
| α, β, γ (°) | 90, 90, 90 |
| Resolution (Å) | 50.00-1.80 (1.83-1.80) * |
| *R*_merge_ | 12.0 (36.2) |
| *I* / σ*I* | 26.4 (11.5) |
| Completeness (%) | 99.0 (100.0) |
| Redundancy | 22.7 (22.7) |
|  |  |
| **Refinement** |  |
| Resolution (Å) | 24.56-1.80 (1.86-1.80) |
| No. reflections | 15058 (1463) |
| *R*_work_ / *R*_free_ | 18.1/18.6 (20.3/22.9) |
| No. atoms |  |
| Protein | 921 |
| Ligand/ion | 14 |
| Water | 140 |
| *B*-factors |  |
| Protein | 21.8 |
| Ligand/ion | 21.7 |
| Water | 36.2 |
| R.m.s. deviations |  |
| Bond lengths (Å) | 0.006 |
| Bond angles (°) | 0.94 |

*Values in parentheses are for the highest-resolution shell; one crystal for each structure.

**NMR Table**

## **Supplementary Table 6.** NMR data of **8** in CD_3_OD^1^.

| Position | *δ*_H_ [ppm] (m, *J*_H_ in Hz) | *δ*_C_ [ppm]^2^ |
| --- | --- | --- |
| 1 | - | 176.5 |
| 2 | 4.33 (1H, dd, 4.8, 7.6) | 54.8 |
| 3 | 1.74 (1H, m)  1.92 (1H, m) | 28.8 |
| 4 | 1.59 (2H, m) | 30.0 |
| 1′ | 2.78 (1H, m) | 34.1 |
| 2′, 6′ | 5.82 (1H, br d, 10.7)  5.85 (1H, br d, 10.1) | 131.0 |
| 3′, 5′ | 5.68 (2H, br d, 10.5) | 124.9 |
| 4′ | 4.88 (1H, m) | 43.1 |
| 7′ | 1.96 (3H, m) | 21.0 |
| 8′ | - | 171.3 |
| 1′′ |  | n.d. |
| 2′′ | 3.52 (1H, m) | 67.1 |
| 3′′ | 1.89 (1H, m) | 36.9 |
| 4′′ | 1.28 (1H, m)  1.63 (1H, m) | 24.9 |
| 5′′ | 0.99 (3H, t, 7.4) | 10.4 |
| 6′′ | 1.03 (3H, d, 6.9) | 13.6 |
| 7′′ | 2.59 (3H, s) | 32.3 |

^1^ Data was collected at 700 MHz for ^1^H NMR and at 175 MHz for ^13^C NMR;

^2^ From 2D spectra;

^3^ n.d., not detected.

## **Supplementary Table 7.** NMR data of α-methyl-KAPA (**5**) in D_2_O^1^.

| Position | *δ*_H_ [ppm] (m, *J*_H_ in Hz) | *δ*_C_ [ppm] |
| --- | --- | --- |
| 1 |  | 184.5 |
| 2 | 2.31 (1H, m) | 40.8 |
| 3 | 1.46 (1H, m)  1.33 (1H, m) | 33.1 |
| 4 | 1.21 (2H, m) | 26.0 |
| 5 | 1.51 (2H, m) | 22.5 |
| 6 | 2.63 (1H, m)  2.55 (1H, m) | 37.8 |
| 7 |  | 209.3 |
| 8 | 4.17 (1H, q, 7.4) | 54.6 |
| 9 | 1.46 (1H, d, 7.4) | 14.5 |
| 10 | 1.00 (1H, d, 6.9) | 16.9 |

^1^ Data was collected at 500 MHz for ^1^H NMR and at 125 MHz for ^13^C NMR.

## **Supplementary Table 8.** NMR data of **9** in CD_3_OD^1^.

| Position | *δ*_H_ [ppm] (m, *J*_H_ in Hz) | *δ*_C_ [ppm] |
| --- | --- | --- |
| 1/1' |  | 181.5 |
| 2/2' | 2.39 (m) | 41.2 |
| 3/3' | 1.68 (m)  1.44 (m) | 35.1 |
| 4/4' | 1.43 (m) | 28.5 |
| 5/5' | 1.66 (m) | 29.9 |
| 6/6' | 2.76 (t, 7.8) | 35.1 |
| 7/7' |  | 153.4 |
| 8/8' |  | 149.6 |
| 9/9' | 2.49 (s) | 20.8 |
| 10/10' | 1.13 (d, 7.0) | 17.9 |

^1^ Data was collected at 500 MHz for ^1^H NMR and at 125 MHz for ^13^C NMR.

## **Supplementary Table 9.** NMR data of α-methyldesthiobiotin (**3**) in D_2_O^1^.

| Position | *δ*_H_ [ppm] (m, *J*_H_ in Hz) | *δ*_C_ [ppm] |
| --- | --- | --- |
| 1 |  | 183.4 |
| 2 | 2.38 (1H, m) | 40.0 |
| 3 | 1.50 (1H, m)  1.38 (1H, m) | 33.0 |
| 4 | 1.25 (2H, m) | 26.4 |
| 5 | 1.27 (1H, m)  1.19 (1H, m) | 25.4 |
| 6 | 1.43 (2H, m) | 28.5 |
| 7 | 3.69 (1H, m) | 56.0 |
| 8 |  | 165.7 |
| 9 | 3.81 (1H, m) | 51.5 |
| 10 | 1.01 (1H, d, 6.6) | 14.1 |
| 11 | 1.03 (1H, d, 7.0) | 16.5 |

^1^ Data was collected at 500 MHz for ^1^H NMR and at 125 MHz for ^13^C NMR.

## **Supplementary Table 10.** NMR data of ANDA (**6**) in D_2_O^1^.

| Position | *δ*_H_ [ppm] (m, *J*_H_ in Hz) | *δ*_C_ [ppm] |
| --- | --- | --- |
| 1 |  | 174.2 |
| 2 | 3.71 (1H, t, 6.2) | 54.2 |
| 3 | 1.96 (2H, m) | 29.5 |
| 4 | 2.27 (2H, m) | 27.8 |
| 5 | 6.18 (1H, dt, 6.8, 15.3) | 141.7 |
| 6 | 6.32 (1H, dd, 10.8, 15.2) | 129.4 |
| 7 | 7.17 (1H, dd, 10.8, 15.4) | 144.9 |
| 8 | 5.85 (1H, d, 15.4) | 121.1 |
| 9 |  | 172.9 |

^1^ Data was collected at 500 MHz for ^1^H NMR and at 125 MHz for ^13^C NMR.

## **Supplementary Table 11.** NMR data of Ala-ANDA (**10**) in CD_3_OD^1^.

| Position | *δ*_H_ [ppm] (m, *J*_H_ in Hz) | *δ*_C_ [ppm] |
| --- | --- | --- |
| 1 |  | 177.4 |
| 2 | 4.29 (1H, dd, 8.2, 4.6) | 55.6 |
| 4 | 1.83 (1H, m)  2.01 (1H, m) | 32.7 |
| 5 | 2.26 (2H, dt, 7.4) | 30.3 |
| 6 | 6.16 (1H, dt, 15.1, 6.8) | 143.9 |
| 7 | 6.26 (1H, dd, 15.2, 10.8) | 130.4 |
| 8 | 7.21 (1H, dd, 15.2, 10.8) | 145.9 |
| 9 | 5.79 (1H, d, 15.3) | 122.0 |
| 1' |  | 171.5 |
| 2' |  | 170.4 |
| 3' | 3.95 (1H, q, 7.0) | 50.3 |

^1^ Data was collected at 500 MHz for ^1^H NMR and at 125 MHz for ^13^C NMR.

## **Supplementary Table 12.** NMR data of (*E*)-**6*** in D_2_O^1^.

| Position | *δ*_H_ [ppm] (m, *J*_H_ in Hz) | *δ*_C_ [ppm] |
| --- | --- | --- |
| 1 |  | 174.3 |
| 2 | 4.15 (1H, m) | 60.3 |
| 3 | 2.46 (1H, m)  2.02 (1H, m) | 28.6 |
| 4 | 2.21 (1H, m)  1.87 (1H, m) | 30.4 |
| 5 | 4.23 (1H, m) | 61.7 |
| 6 | 5.70 (1H, dd, 7.9, 15.6) | 127.3 |
| 7 | 6.00 (1H, dt, 7.2, 15.3) | 130.0 |
| 8 | 3.17 (3H, d, 6.9) | 37.2 |
| 9 |  | 176.3 |

^1^ Data was collected at 500 MHz for ^1^H NMR and at 125 MHz for ^13^C NMR.

## **Supplementary Table 13.** NMR data of (*Z*)-(**6***) in D_2_O^1^.

| Position | *δ*_H_ [ppm] (m, *J*_H_ in Hz) | *δ*_C_ [ppm] |
| --- | --- | --- |
| 1 |  | 174.4 |
| 2 | 4.15 (1H, dd, 9.6, 4.7) | 61.1 |
| 3 | 2.34 (1H, m)  2.21 (1H, m) | 28.7 |
| 4 | 2.20 (1H, m)  1.74 (1H, m) | 30.0 |
| 5 | 4.46 (1H, dt, 9.6, 6.7) | 57.2 |
| 6 | 5.73 (1H, dd, 10.8, 9.7) | 126.8 |
| 7 | 5.94 (1H, dt, 10.8, 7.7) | 129.2 |
| 8 | 3.21 (2H, dd, 7.8, 3.2) | 33.4 |
| 9 |  | 176.5 |

^1^ Data was collected at 500 MHz for ^1^H NMR and at 125 MHz for ^13^C NMR.

## **Supplementary Table 14.** NMR data of acidomycin (**1**) in CD_3_OD^1^.

| Position | *δ*_H_ [ppm] (m, *J*_H_ in Hz) | *δ*_C_ [ppm] |
| --- | --- | --- |
| 1 |  | 177.8 |
| 2 | 2.29 (2H, t, 7.4) | 35.0 |
| 3 | 1.62 (2H, m) | 26.1 |
| 4 | 1.39 (2H, m) | 29.9 |
| 5 | 1.42 (2H, m) | 26.1 |
| 6 | 1.85 (1H, m)  1.69 (1H, m) | 39.7 |
| 7 | 4.76 (1H, m) | 59.2 |
| 8 | 3.51 (1H, dd, 15.7, 1.8)  3.48 (1H, dd, 15.7, 0.9) | 32.9 |
| 9 |  | 177.1 |

^1^ Data was collected at 500 MHz for ^1^H NMR and at 125 MHz for ^13^C NMR.

**References**

1. Li B, Yin F, Zhao X, et al. Colistin Resistance Gene mcr-1 Mediates Cell Permeability and Resistance to Hydrophobic Antibiotics. *Front Microbiol*. 2019;10:3015. https://doi:10.3389/fmicb.2019.03015.

2. Robert A. Copeland, ed. *Enzymes: A Practical Introduction to Structure, Mechanism, and Data Analysis*. Wiley-VCH, A JOHN WILEY & SONS, INC., PUBLICATION; 2000.

3. Laskowski RA, Swindells MB. LigPlot+: multiple ligand-protein interaction diagrams for drug discovery. *J Chem Inf Model*. 2011;51(10):2778-2786. https://doi:10.1021/ci200227u.

4. Ditta G, Stanfield S, Corbin D, Helinski DR. Broad host range DNA cloning system for gram-negative bacteria: construction of a gene bank of Rhizobium meliloti. *Proc Natl Acad Sci U S A*. 1980;77(12):7347-7351. https://doi:10.1073/pnas.77.12.7347.

5. Kieser T. *Practical streptomyces genetics*. Norwich: John Innes Foundation; 2000.

6. Fu J, Teucher M, Anastassiadis K, Skarnes W, Stewart AF. A recombineering pipeline to make conditional targeting constructs. *Methods Enzymol*. 2010;477:125-144. https://doi:10.1016/S0076-6879(10)77008-7.

7. Wang H, Li Z, Jia R, et al. RecET direct cloning and Redαβ recombineering of biosynthetic gene clusters, large operons or single genes for heterologous expression. *Nat Protoc*. 2016;11(7):1175-1190. https://doi:10.1038/nprot.2016.054.

8. Liu H, Naismith JH. A simple and efficient expression and purification system using two newly constructed vectors. *Protein Expr Purif*. 2009;63(2):102-111. https://doi:10.1016/j.pep.2008.09.008.

9. Myronovskyi M, Rosenkränzer B, Nadmid S, Pujic P, Normand P, Luzhetskyy A. Generation of a cluster-free Streptomyces albus chassis strains for improved heterologous expression of secondary metabolite clusters. *Metab Eng*. 2018;49:316-324. https://doi:10.1016/j.ymben.2018.09.004.

10. Shirling EB, Gottlieb D. Cooperative description of type cultures of Streptomyces. IV. Species descriptions from the second, third and fourth studies. *International Journal of Systematic Bacteriology*. 1969;19(4):391-512. https://doi:10.1099/00207713-19-4-391.

11. Xie F, Zhao H, Liu J, et al. Autologous DNA mobilization and multiplication expedite natural products discovery from bacteria. *Science*. 2024;386(6727):eabq7333. https://doi:10.1126/science.abq7333.
